# CD103^+^ T Cells Eliminate Damaged Alveolar Epithelial Type II Cells Under Oxidative Stress to Prevent Lung Tumorigenesis

**DOI:** 10.1002/advs.202503557

**Published:** 2025-05-08

**Authors:** Yu Xu, Haorui Luo, Jiahao Wang, Haifeng Liu, Luonan Chen, Hongbin Ji, Zimu Deng, Xiaolong Liu

**Affiliations:** ^1^ Key Laboratory of Multicellular Systems CAS Center for Excellence in Molecular Cell Science Shanghai Institute of Biochemistry and Cell Biology University of Chinese Academy of Sciences Chinese Academy of Sciences 320 Yueyang Road Shanghai 200031 China; ^2^ Zhongshan Institute for Drug discovery Shanghai Institute of Materia Medica Chinese Academy of Sciences Zhongshan 528400 China; ^3^ Key Laboratory of Systems Health Science of Zhejiang Province School of Life Science Hangzhou Institute for Advanced Study University of Chinese Academy of Sciences Chinese Academy of Sciences Hangzhou 310024 China; ^4^ School of Life Science and Technology ShanghaiTech University 319 Yueyang Road Shanghai 200031 China

**Keywords:** AT2 cell, CD103^+^ T cell, lung adenocarcinoma, MED23, oxidative stress, tumorigenesis

## Abstract

The nexus between aging‐associated immune deteriorations and tumorigenesis of lung cancers remains elusive. In a mouse model with *Med23* depletion in T cells (*Med23* ^−/−^), it is found a strong association between the decline of CD103^+^ T cells and spontaneous alveolar epithelial type II cell (AT2 cell)‐originated lung adenocarcinomas. The reduction of CD103^+^ T cells in the lung results in an accumulation of AT2 cells bearing oxidative damages, which appears to be the major origin of the lung adenocarcinoma. Functional experiments reveal CD103^+^ T cells can eradicate oxidative‐damage‐bearing AT2 cells as well as ROS‐dependent, KRAS (G12D)‐driven tumorigenesis. In vitro co‐cultures prove CD103^+^ T cells, especially CD103^+^ CD8^+^ T cells, exhibit a killing capacity that matches the oxidative stress level in the target cells. In aged animals, it is found the abundance of CD103^+^ CD8^+^ T cells in the lung declines with age, accompanied by an accumulation of oxidative‐damage‐bearing AT2 cells. Collectively, the study establishes the vital function of CD103^+^ T cells in surveilling epithelial cells under oxidative stress to prevent malignancies, and unravels a potential immuno‐dysregulation in the aged lung which contributes to tumorigenesis.

## Graphical abstract

This work unravels a novel function of CD103^+^ T cells in eradicating oxidative‐stressed somatic cells to prevent lung tumorigenesis and identifies specific CD103^+^ T cell‐decline as a key feature in the aged lung. This comprehensive study highlights the contribution of immuno‐dysregulation to the high incidence of aging‐associated lung cancers.

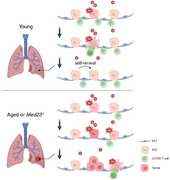

## 1 Introduction

Tissue‐resident memory T (T_RM_) cells represent a unique T cell population that exhibits long‐term persistence in non‐lymphoid tissues. To establish local residency, T_RM_ progenitors gradually acquire distinct functional and migratory features such as: upregulation of molecules for tissue retention such as CD69 and CD103 and downregulation of tissue egress molecules such as S1PR1 and CCR7.^[^ ^1^ ^]^ Various studies prove that T_RM_ cells are more efficient protecting tissue from recurrent infections as compared to memory T cells in circulation, underscoring the importance of T_RM_ cells in regional immune response. In addition to protecting against pathogens, the abundance of tumor infiltrating T_RM_ cells appears to associate with improved patient survival in multiple type of solid tumors, suggesting a central role for T_RM_ cells in anti‐tumor immunity.^[^ ^1^, ^2^ ^]^ Thus, the detailed function of T_RM_ cells during tumor initiation and progression deserves in‐depth investigation.

Cellular oxidative stress acts as an essential player in cancer initiation and progression.^[^ ^3^, ^4^ ^]^ The reactive oxygen species (ROS) can modify proteins, lipids, DNA and RNA, causing damages to these macromolecules. The accumulated oxidative damages and subsequent oncogenic alterations rendering growth advantages to somatic cells are considered key pro‐tumorigenic events.^[^ ^5^, ^6^ ^]^ In addition to the substantial evidence showing a correlation between oxidative stress and tumorigenesis from clinical studies, the spontaneous malignancies in mice lacking antioxidant enzymes (e.g. Prdx1) fully demonstrate the functional role of oxidative stress in initiating cancerous transformation.^[^ ^7^, ^8^ ^]^ Besides their toxicities, ROS serve as signaling molecules facilitating cancer cell survival, proliferation, metabolism, invasion and metastasis.^[^ ^9^ ^]^ High level of ROS in tumor microenvironment appears to limit the cytotoxicity of NK cells and T cells^[^ ^10^, ^11^, ^12^ ^]^ but enhances the suppressive function of the myeloid‐derived suppressor cells (MDSCs),^[^ ^13^ ^]^ thereby establishing a microenvironment favoring tumor immune escape. In spite of the many pro‐tumorigenic features of oxidative stress, dietary antioxidants in clinical trials fail to prevent several types of cancer,^[^ ^14^, ^15^ ^]^ emphasizing the intricacies of redox biology. Indeed, subsequent studies proved that ROS can suppress cancer by triggering cellular senescence and cell death such as ferroptosis, which antagonize certain steps of tumorigenesis.^[^ ^16^, ^17^ ^]^ Likewise, it has been observed that cancer cells actively upregulate the antioxidant capacity to control intracellular ROS and to maintain redox homeostasis.^[^ ^18^ ^]^ Taken together, existing knowledge reveals the pleiotropic features of oxidative stress in cancer, and the full landscape of the oxidative stress‐related responses in cancer biology needs further exploration.

Our previous studies reported that loss of MED23, one subunit of the tail module of the mediator complex, enhances anti‐tumor function of conventional T cells in PyMT transgenic mice, but inhibits anti‐tumor activity of invariant natural killer T (iNKT) cells in B16F10 melanoma lung metastasis mouse model.^[^ ^19^, ^20^ ^]^ Here we find loss of MED23 in T cells (*Med23* ^−/−^) reduces CD103^+^ T cells within tissues including the lung. Interestingly, *Med23* ^−/−^ mice spontaneously develop tumors within multiple organs, with alveolar epithelial type II cell (AT2 cell)‐originated lung adenocarcinoma as a major manifestation. The major pro‐tumorigenic step in the AT2 cells is mapped to the oxidative stress. By adoptive transfers and in vitro cocultures, we show that in the lung, CD103^+^ T cells eliminate damaged AT2 cells under oxidative stress to prevent carcinogenesis. This study provides novel insights into the regional immune regulations against malignancies arising from oxidative stress and underscores the importance of CD103^+^ T cells in maintaining tissue homeostasis. More importantly, we observed a decline of CD103^+^ T cells and an accumulation of oxidative‐damage‐bearing AT2 cells in the aged lung, suggesting the decline of CD103^+^ T might represent a key feature of the aged T cell compartment contributing to the higher incidence of lung cancers in the aged population.^[^ ^21^, ^22^ ^]^

## 2 Result

### 2.1 Mice with *Med23* Deletion in T Cells Develop AT2 Cell‐Originated Lung Adenocarcinoma

**Figure 1:**
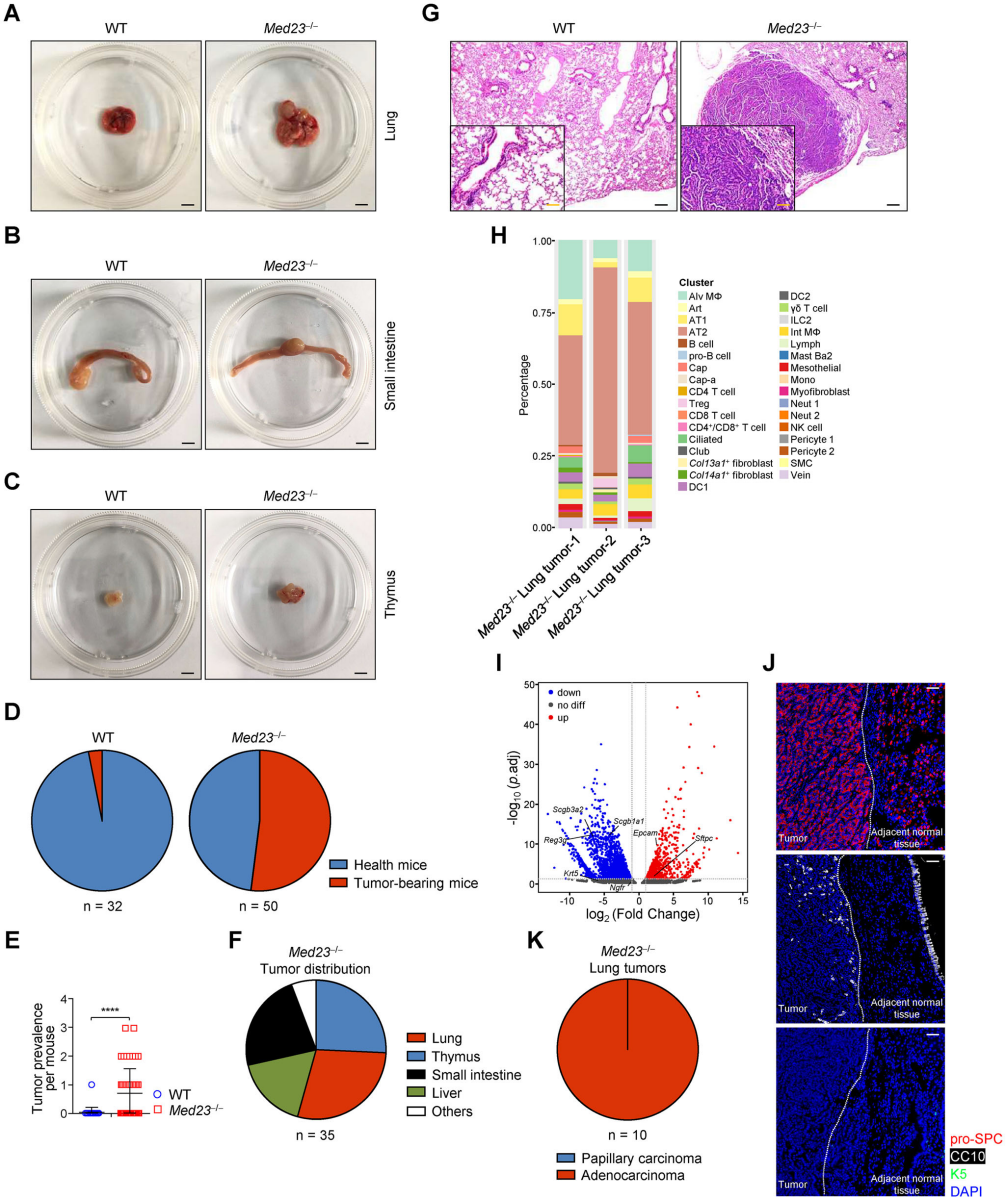
AT2 cell‐derived lung adenocarcinoma is developed in *Med23* ^−/−^ aged mice. A–C) Spontaneous tumors of the lung A), small intestine B) and thymus C) in *Med23* ^−/−^ aged mice. Scale bar, 600 mm. D) The occurrence of spontaneous tumors in WT and *Med23* ^−/−^ aged mice (WT: *n* = 32 mice; *Med23* ^−/−^: *n* = 50 mice). E) The tumor prevalence per mouse in WT and *Med23* ^−/−^ aged mice (WT: *n* = 32 mice; *Med23* ^−/−^: *n* = 50 mice). F) The tumor distribution in *Med23* ^−/−^ aged mice (*n* = 35 mice). G) Hematoxylin and eosin (H&E)‐stained lung sections in WT and *Med23* ^−/−^ aged mice. Scale bar: black, 200 µm; orange, 100 µm. H) The CIBERSORTx analysis of the transcriptional landscapes from *Med23* ^−/−^ lung tumors and lung single‐cell. The full name of cell populations in the lungs is performed in Table S1 (Supporting Information). I) Volcano plot of gene expression in lung tumors and adjacent normal tissues of *Med23* ^−/−^ aged mice. Grey vertical and horizontal lines represent the filtering criteria: | log_2_ (Fold Change) | = 1.0 and adjust *p*‐value = 0.05. J) Representative immunofluorescence staining for pro‐SPC, CC10, and K5 in lungs of *Med23* ^−/−^ aged mice. Scale bar: 50 µm. K) The probability of adenocarcinoma and papillary carcinoma in *Med23* ^−/−^ lung tumors (*n* = 10 mice). The data (E) are presented as the mean ± s.d. For all panels: ^****^ *p* < 0.0001 by Student's *t*‐test. All data are representative of (A–C, G, J) or combined from (D–F, H, I, K) at least three independent experiments.

We had generated *Cd4*‐Cre‐drived *Med23* conditional knockout mice (designated KO mice) to demonstrate the roles of MED23 in regulating the differentiation and function of conventional T cells and iNKT cells.^[^ ^19^, ^20^ ^]^ To study whether the dysfunctional T cells in these mice alter tissue fitness, we analyzed aged *Med23* ^−/−^ mice for diseases. Unexpectedly, we found *Med23* ^−/−^ mice spontaneously developed tumors at multiple organs by the age of 18 months (**Figure** **1**A–D). A similar tendency of tumor development was observed in both male and female *Med23* ^−/−^ mice (Figure S1A,B, Supporting Information). Moreover, the tumor prevalence per mouse was significantly higher in *Med23* ^−/−^ mice than in wild type (WT) littermates (Figure 1E). When we analyzed the tumor distributions in these mice, we found that the lung, the liver, the small intestine and the thymus were mostly prone to tumor development (Figure 1F). The existence of malignant cells within the tumors were further confirmed by histology analysis (Figure 1G; Figure S2A–C, Supporting Information). Overall, these results indicated that loss of MED23 in T cells leads to spontaneous tumorigenesis in aged mice.

Since the lung appeared to be one of the major organs bearing tumors in the *Med23* ^−/−^ mice (Figure 1F), we next focused on the lung to unravel the relationship between T cell dysfunction, tissue fitness and tumor initiation. We first investigated the origin and the subtype of the lung tumors in *Med23* ^−/−^ mice by applying the CIBERSORTx analysis to characterize the cell composition of the *Med23* ^−/−^ lung tumors based on their gene expression profiles, and found that *Med23* ^−/−^ lung tumors exhibited signatures similar to AT2 cells (Figure 1H; Table S1, Supporting Information).^[^ ^23^ ^]^ *Sftpc*, the gene predominantly expressed in AT2 cells, was markedly upregulated in *Med23* ^−/−^ lung tumors compared to that in *Med23* ^−/−^ normal lung tissues. Instead, the markers for club cells, bronchioalveolar stem cells and basal cells, which represent the other candidate origins of lung tumors, such as *Scgb1a1* and *Krt5* either decreased or maintained their normal expression in *Med23* ^−/−^ lung tumors (Figure 1I,J).^[^ ^24^, ^25^, ^26^ ^]^ Moreover, *Med23* ^−/‐ ^lung tumors primarily exhibited histologic phenotype of adenocarcinoma instead of papillary carcinoma which has a high number in the CC10^+^ cells‐derived lung cancer (Figure 1G,K).^[^ ^25^ ^]^ Taken together, these results largely demonstrated that *Med23* ^−/−^ lung tumor is the AT2 cell‐derived lung adenocarcinoma. To exclude the direct influence of *Cd4*‐*Cre*‐drived *Med23* knockout in AT2 cells, we analyzed *Med23* expression and found similar *Med23* mRNA levels in WT and *Med23* ^−/−^ AT2 cells (Figure S3, Supporting Information).

### 2.2 Mutation Rate of Oncogenes is Increased in the Spontaneous Lung Adenocarcinoma

**Figure 2:**
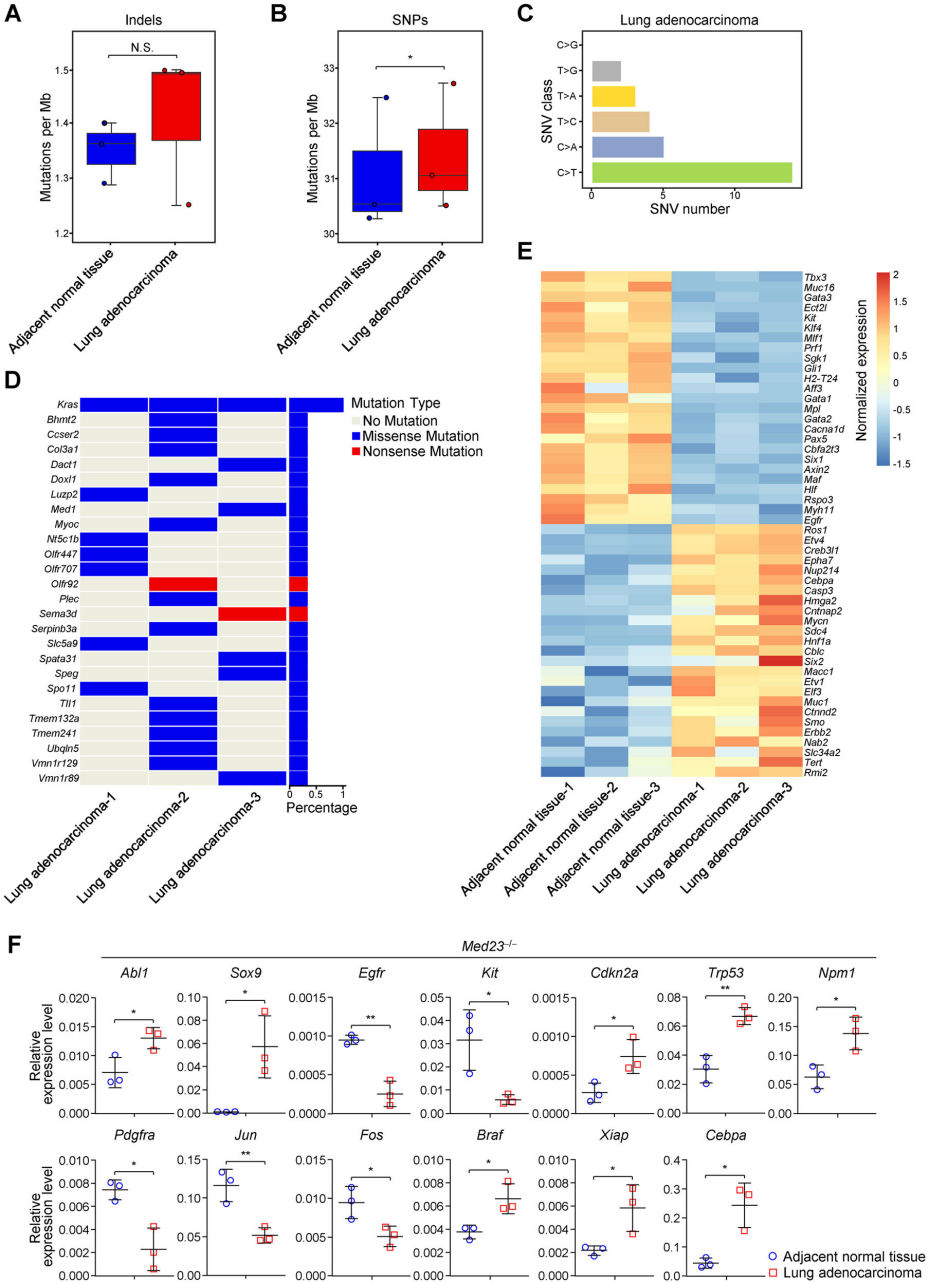
The characteristics of spontaneous lung adenocarcinoma in *Med23* ^−/−^ aged mice. A,B) The boxplots displaying the insertion‐deletion (Indel) A) and the SNP B) per Mb of lung adenocarcinomas and adjacent normal tissues in *Med23* ^−/−^ aged mice (*n* = 3 mice, paired *t*‐test). C) The analysis of non‐synonymous single‐nucleotide variant (SNV) in lung adenocarcinomas from *Med23* ^−/−^ aged mice. D) The heatmap displaying oncogene mutation in lung adenocarcinomas from *Med23* ^−/−^ aged mice. E) The expression of top 50 deferentially expressed genes with known cancer driver genes between lung adenocarcinomas and adjacent normal tissues from *Med23* ^−/−^ aged mice. Absolute expression values were transformed into *Z* scores before visualization. Filtering criteria: | log_2_ (Fold Change) | > 1 and adjust *p*‐value < 0.05. F) Quantitative RT‐PCR analysis of *Abl1*, *Sox9*, *Egfr*, *Kit*, *Cdkn2a*, *Trp53*, *Npm1*, *Pdgfra*, *Jun*, *Fos*, *Braf*, *Xiap* and *Cebpa* mRNA levels in lung adenocarcinomas and adjacent normal tissues of *Med23* ^−/−^ aged mice (*n* = 3 mice). All expression levels were normalized to *Actb* expression. The data (F) are presented as the mean ± s.d. For all panels: ^*^ *p* < 0.05; ^**^ *p* < 0.01 by Student's *t*‐test; N.S.: no significance. All data are combined from (A‐F) at least three independent experiments.

To further characterize the lung adenocarcinomas in *Med23*‐deficient mice, we performed whole exome sequencing and found that *Med23* ^−/−^ lung adenocarcinomas displayed increased single nucleotide polymorphism (SNP) mutation rate compared with those of *Med23* ^−/−^ normal tissue controls (**Figure** **2**A,B). These results encouraged us to explore the mutation signatures of the *Med23* ^−/−^ lung adenocarcinomas. Interestingly, we found that lung adenocarcinomas of *Med23* ^−/−^ mice exhibited distinct types of non‐synonymous mutations (Figure 2C), such as the C to T conversion related to transcription and mismatch repair deficiency,^[^ ^27^, ^28^ ^]^ the C to A conversion related to the oxidative damage,^[^ ^29^ ^]^ and the T to C conversion related to aberrant base excision repair.^[^ ^30^ ^]^ Moreover, high frequencies of non‐synonymous mutations were detected in multiple oncogenes, such as *Kras* and *Col3a1*, which are intimately associated with lung adenocarcinoma development (Figure 2D).^[^ ^31^, ^32^ ^]^ Transcriptional levels of genes related to cancer development were also significantly changed in *Med23* ^−/−^ lung adenocarcinomas (Figure 2E,F).^[^ ^33^, ^34^ ^]^ Overall, our data indicated that AT2 cell‐derived lung adenocarcinomas in *Med23* ^−/−^ mice are largely driven by accumulation of oncogenic mutations.

### 2.3 Accumulation of AT2 Cells with Oxidative Damages in *Med23* ^−/−^ Mice Largely Initiates Adenocarcinoma

**Figure 3:**
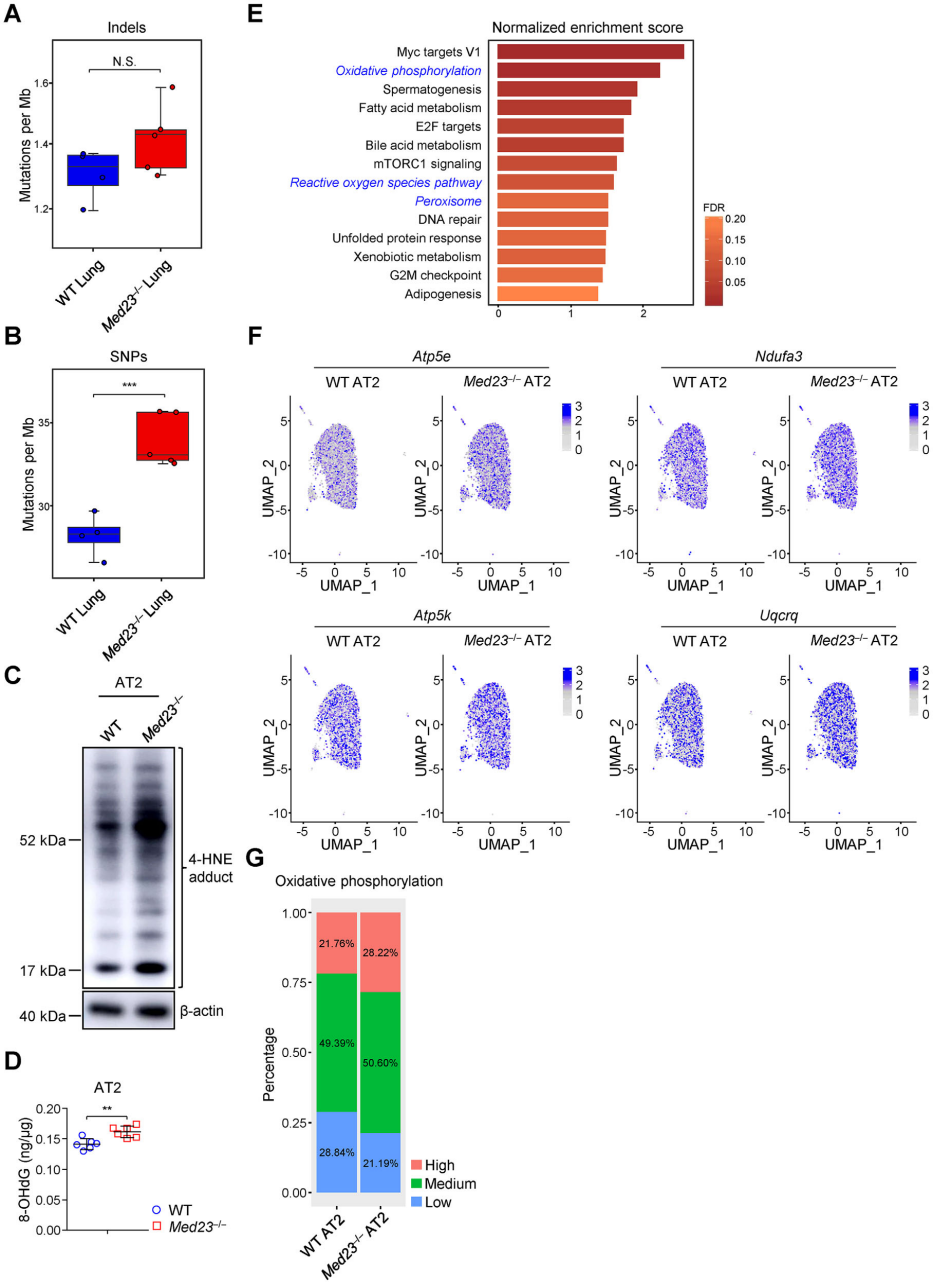
An increased accumulation of AT2 cells bearing oxidative stress and oxidative DNA damage in *Med23* ^−/−^ mice. A,B) The boxplots displaying the Indel A) and the SNP B) per Mb of normal lung tissues from WT and *Med23* ^−/−^ aged mice (WT: *n* = 4 mice; *Med23* ^−/−^: *n* = 5 mice). C) Immunoblot analysis of 4‐HNE in isolated WT and *Med23* ^−/−^ AT2 cells. D) Elisa of 8‐OHdG expression in isolated WT and *Med23* ^−/−^ AT2 cells (*n* = 6 mice). E) The enrichment analysis of hallmark gene sets in WT and *Med23* ^−/−^ AT2 cell gene expression (*n* = 3 mice). Blue and italic: categories related to oxidative stress. F) Single‐cell RNA‐seq analysis of *Atp5e*, *Ndufa3*, *Atp5k* and *Uqcrq* expression in WT and *Med23* ^−/−^ AT2 cells. G) The percentage of AT2 cells with low (AUCell score ≤ 0.33), medium (AUCell score > 0.33 and < 0.38) and high (AUCell score ≥ 0.38) expression levels of genes related to oxidative phosphorylation in WT and *Med23* ^−/−^ AT2 cells (WT: *n* = 7756 cells; *Med23* ^−/−^: *n* = 7807 cells). The gene expression related to oxidative phosphorylation was evaluated by “GOBP_OXIDATIVE_PHOSPHORYLATION” pathway using AUCell v1.12.0. The upper or lower quartile value of AUCell scores of gene expression related to oxidative phosphorylation from all AT2 cells is determined as the threshold of high or low expression level. The data (D) are presented as the mean ± s.d. For all panels: ^**^ *p* < 0.01; ^***^ *p* < 0.001 by Student's *t*‐test; N.S.: no significance. The data are combined from (A, B, E) two independent experiments. The data are representative of (C) or combined from (D) at least three independent experiments. The data are combined from (F,G) one independent experiment.

To determine whether *Med23* ^−/−^ T cells impact mutation accumulation before lung adenocarcinoma initiation, we examined the mutation rate of normal lung tissues in WT and *Med23* ^−/−^ aged mice. As shown in **Figure** **3**A,B, *Med23* ^−/−^ lungs had a higher SNP mutation rate than that of WT controls and a similar trend in the insertion‐deletion (Indel) mutation rate was observed as well. γ‐H2AX, a sensor of DNA damage, was found significantly increased in *Med23* ^−/−^ lungs (Figure S4A, Supporting Information).^[^ ^35^ ^]^ When we analyzed the types of DNA damage in the lung by HPLC‐MS, we found the content of 8‐hydroxy‐2' ‐deoxyguanosine (8‐OHdG) which is a biomarker of oxidative DNA damage and a putative driver of carcinogenesis, showed a slightly increase in *Med23* ^−/−^ lungs compared with that of WT controls (Figure S4B, Supporting Information).^[^ ^29^ ^]^ Meanwhile, the alkylation and deamination of DNA in *Med23* ^−/−^ lungs stayed at the same levels as in WT lungs (Figure S4B, Supporting Information). These results implied that loss of MED23 in T cells somehow give rise to increased oxidative DNA damages in the lung which contributes to the oncogenic mutations and AT2 cell‐derived adenocarcinomas. When we compared the mutation signatures from *Med23* ^−/−^ lung adenocarcinoma with that from a mouse model induced by tobacco carcinogen,^[^ ^36^ ^]^ which is the classic model for oxidative stress‐induced tumorigenesis,^[^ ^37^ ^]^ we found similarities between the two, such as: increased C > T mutations and high frequencies of non‐synonymous mutations in *Kras* (Figure 2C,D).^[^ ^36^ ^]^ These data indicate that although oxidative stress can initiate mutations in somatic cells, the mutation load in malignant cells depends on mutation rate, selection and recombination rate.^[^ ^38^, ^39^ ^]^ To further characterize the mechanism underlying AT2 cell transformation, we purified AT2 cells from adult mice (6 months old) and quantified the cellular adducts of 4‐hydroxy‐2‐nonenal (4‐HNE), a product of lipid peroxidation and a biomarker of oxidative stress.^[^ ^40^ ^]^ Western blot revealed that the level of 4‐HNE in AT2 cells from *Med23* ^−/−^ lungs was markedly higher than that of WT controls (Figure 3C). Then, we assessed the level of oxidative DNA damage in AT2 cells and found 8‐OHdG was also increased in *Med23* ^−/−^ AT2 cells (Figure 3D). Having confirmed the oxidative stress and oxidative DNA damages in *Med23* ^−/−^ AT2 cells, we sought to investigate the potential origin of the excessive free radicals in *Med23* ^−/−^ lungs. Given neutrophils and macrophages are considered the major cellular sources of ROS around somatic cells,^[^ ^41^, ^42^ ^]^ we first measured their ROS levels in the lung from WT and *Med23* ^−/−^ adult mice and found that *Med23* deletion in T cells did not influence the ROS production by neutrophils and macrophages (Figures S5A and S6A,B, Supporting Information). We then compared the overall transcriptional changes of AT2 cells in WT and *Med23* ^−/−^ adult mice searching for intrinsic alterations which might account for the oxidative stress prior to tumorigenesis. Gene set enrichment analysis (GSEA) of differentially expressed genes revealed that several pathways related to the oxidative stress were significantly changed in *Med23* ^−/−^ AT2 cells such as oxidative phosphorylation (OXPHOS), one of the major pathways for ROS biogenesis (Figure 3E). Since oxidative stress is correlated with cell cycle arrest and cell proliferation regulates tumorigenesis,^[^ ^43^, ^44^ ^]^ we analyzed the AT2 cell proliferation and found that a similar percentage of EdU^+^ cells among WT and *Med23* ^−/−^ AT2 cells (Figure S7A,B, Supporting Information). To further uncover the mechanism by which *Med23* ^−/−^ T cells lead to oxidative stressed AT2 cells, we subjected purified AT2 cells to single‐cell RNA sequencing analysis. We first observed that AT2 cells from *Med23* ^−/−^ lung displayed similar distribution pattern compared with WT AT2 cells in the unsupervised clustering, implying that loss of MED23 in T cells unlikely leads to systemic alterations in AT2 transcriptome (Figure 3F). When we looked into the expression pattern of genes relating to OXPHOS, we noticed among *Med23* ^−/−^ AT2 cells, there were more cells displaying high expression featuring OXPHOS genes (Figure 3F; Figure S8, Supporting Information). Calculations revealed that the majority *Med23* ^−/−^ and WT AT2 cells had a medium expression of OXPHOS‐related genes, whereas a small portion of the *Med23* ^−/−^ AT2 cells exhibited high OXHPOS gene expression (Figure 3G). These results supported that loss of MED23 in T cells results in the accumulation of certain AT2 cells bearing oxidative DNA damage rather than upregulating oxidative DNA damage globally in AT2 cells. Taken together, our results implied that MED23 deficiency in T cells facilitates the accumulation of AT2 cells bearing oxidative stress and damage.

**Figure 4:**
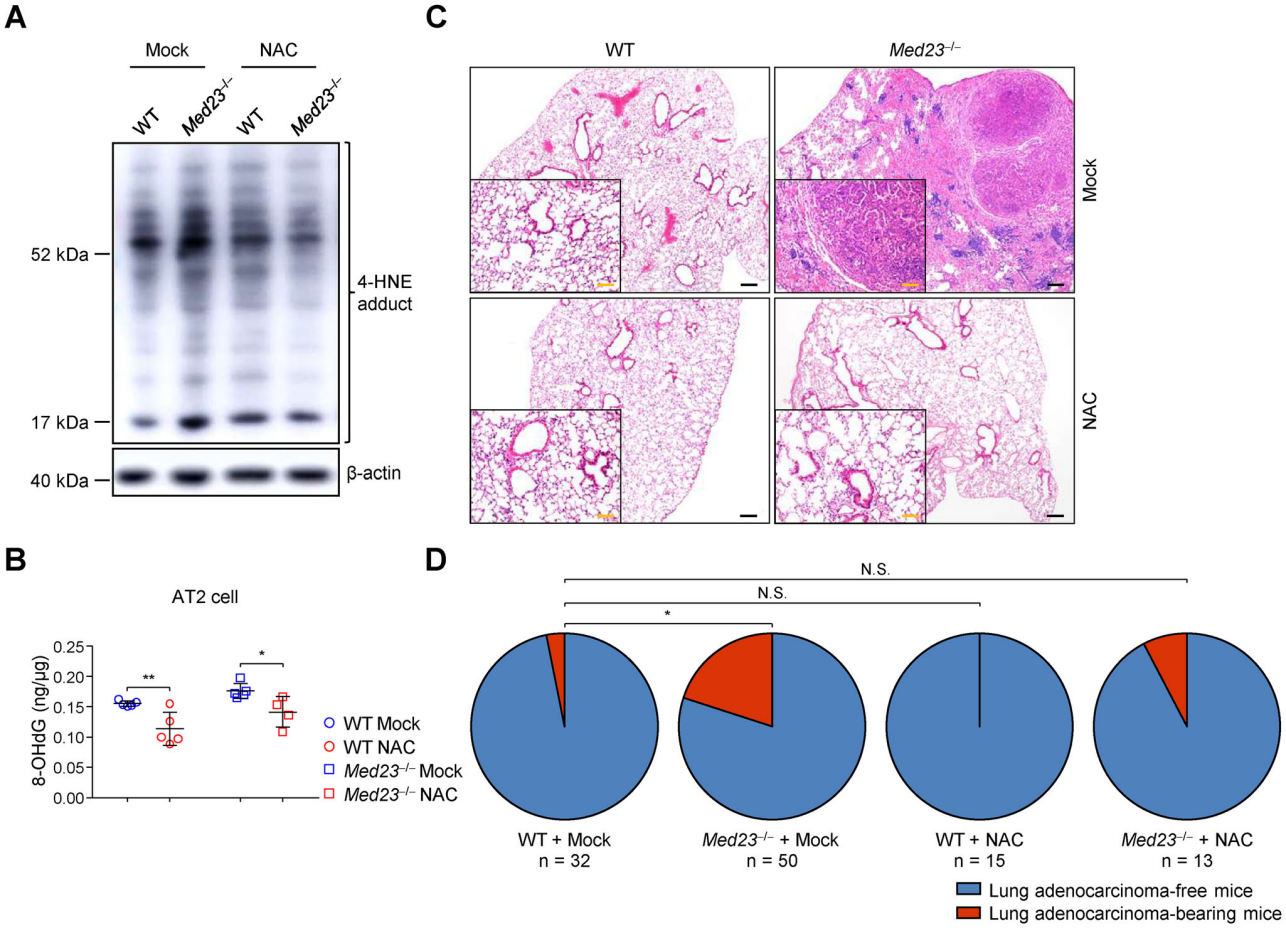
Spontaneous lung adenocarcinoma in *Med23* ^−/−^ aged mice is partially rescued by antioxidant NAC treatment. A,B) WT and *Med23* ^−/−^ mice were given either mock or NAC supplemented in the drinking water from 2‐month‐old and sacrificed 4 months after treatment. AT2 cells were isolated and their 4‐HNE A) and 8‐OHdG expression B) were analyzed (WT and *Med23* ^−/−^ Mock: *n* = 5 mice; *Med23* ^−/−^ NAC: *n* = 4 mice). C,D) WT and *Med23* ^−/−^ mice were given either mock or NAC supplemented in the drinking water from 12‐month‐old. After 6 months of treatment, lungs were harvested for H&E‐stained lung sections C) and spontaneous lung adenocarcinomas D) were analyzed (WT Mock: *n* = 32 mice; *Med23* ^−/−^ Mock: *n* = 50 mice; WT NAC: *n* = 15 mice; *Med23* ^−/−^ NAC: *n* = 13 mice). Scale bar: black, 200 µm; orange, 100 µm. The data B) are presented as the mean ± s.d. For all panels: ^*^ *p* < 0.05; ^**^ *p* < 0.01 by two‐way ANOVA with Bonferroni posthoc test (B) or Fisher's exact test (D); N.S.: no significance. All data are representative of (A,C) or combined from (B,D) at least three independent experiments.

To decipher the connection between cellular oxidative damage and tumorigenesis, we treated a cohort of WT and *Med23* ^−/−^ mice with N‐acetylcysteine (NAC). As a well‐known ROS scavenger, NAC treatment significantly reduced 4‐HNE and 8‐OHdG in AT2 cells (**Figure** **4**A,B). Histology analysis revealed that inhibiting ROS and alleviating oxidative stress in AT2 cells by NAC markedly reduced the occurrence of lung adenocarcinoma in *Med23* ^−/−^ mice (Figure 4C,D). Altogether, these data proved that certain AT2 cells with excessive oxidative stress give rise to lung adenocarcinoma in *Med23* ^−/−^ mice.

### 2.4 Loss of MED23 Downregulates CD103^+^ T Cell Generation in the Lung

**Figure 5:**
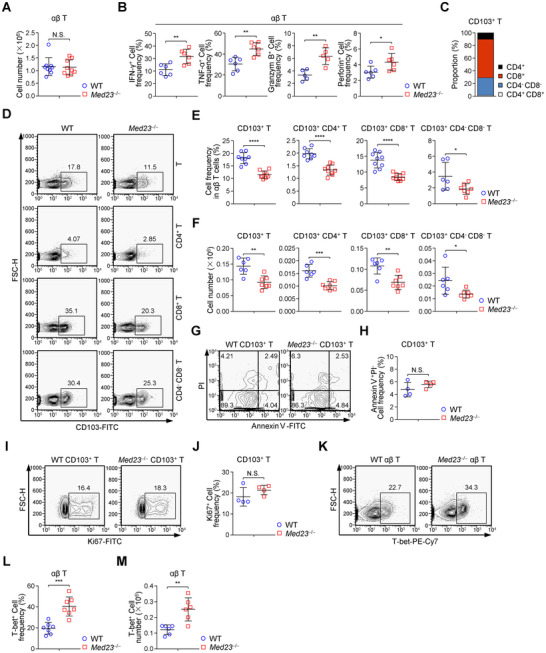
MED23 deficiency impairs CD103^+^ T cell generation in the lungs. A) The absolute number of αβ T cells in WT and *Med23* ^−/−^ lungs (*n* = 9 mice). B) The percentage of IFN‐γ^+^ cells, TNF‐α^+^ cells, Granzym B^+^ cells and Perforin^+^ cells among αβ T cells in lungs from WT and *Med23* ^−/−^ mice after PMA and ionomycin treatment in the presence of brefeldin A for 3.5 h (IFN‐γ, TNF‐α and Perforin: *n* = 6 mice; Granzym B: *n* = 5 mice). C) The proportion of CD4^+^ cells, CD8^+^ cells, CD4^−^ CD8^−^ cells, and CD4^+^ CD8^+^ cells in WT CD103^+^ T cells. D) Flow cytometric analysis of CD103 expression in T cells, CD4^+^ T cells, CD8^+^ T cells and CD4^−^ CD8^−^ T cells from WT and *Med23* ^−/−^ lungs. E,F) The percentage E) and cell number F) of CD103^+^ T cells, CD103^+^ CD4^+^ T cells, CD103^+^ CD8^+^ T cells, and CD103^+^ CD4^−^ CD8^−^ T cells in WT and *Med23* ^−/−^ lungs (percentage: WT CD103^+^ T and WT CD103^+^ CD4^+^ T, *n* = 8 mice, *Med23* ^−/−^ CD103^+^ T and *Med23* ^−/−^ CD103^+^ CD4^+^ T, *n* = 9 mice, WT and *Med23* ^−/−^ CD103^+^ CD8^+^ T, *n* = 9 mice, WT CD103^+^ CD4^−^ CD8^−^ T, *n* = 6 mice, *Med23* ^−/−^ CD103^+^ CD4^−^ CD8^−^ T, *n* = 7 mice; cell number: WT, *n* = 6 mice, *Med23* ^−/−^, *n* = 7 mice). G) Survival of CD103^+^ T cells from WT and *Med23* ^−/−^ lungs was assessed by flow cytometric analysis of Annexin V and PI staining. H) The frequency of Annexin V^+^ PI^−^ cells among CD103^+^ T cells in lungs from WT and *Med23* ^−/−^ mice (*n* = 4 mice). I) Proliferation of CD103^+^ T cells from WT and *Med23* ^−/−^ lungs was assessed by flow cytometric analysis of Ki67 staining. J) The frequency of Ki67^+^ cells among CD103^+^ T cells in lungs from WT and *Med23* ^−/−^ mice (*n* = 4 mice). K) Flow cytometric analysis of T‐bet^+^ cells in WT and *Med23* ^−/−^ αβ T cells from lungs. L,M) The percentage L) and cell number M) of T‐bet^+^ αβ T cells in WT and *Med23* ^−/−^ lungs (percentage: *n* = 7 mice; cell number: *n* = 6 mice). The data (A, B, E, F, H, J, L, M) are presented as the mean ± s.d. For all panels: ^*^ *p* < 0.05; ^**^ *p* < 0.01; ^***^ *p* < 0.001; ^****^ *p* < 0.0001 by Student's *t*‐test; N.S.: no significance. All data are representative of (C, D, G, I, K) or combined from (A, B, E, F, H, J, L, M) at least three independent experiments.

Having established a strong connection between oxidative stress within AT2 cells and tumorigenesis, we sought to investigate the immune‐dysregulation underlying the accumulation of AT2 cells bearing oxidative DNA damage in *Med23* ^−/−^ adult mice. Flow analysis of immune cells within the lung displayed that loss of MED23 in T cells did not alter the cell counts of the major immune cell types such as αβ T cells, NK cells, neutrophils, macrophages, DCs, and B cells (**Figure** **5**A; Figures S5A–C and S9, Supporting Information). Moreover, loss of MED23 causes upregulated anti‐tumor cytokines including interferon‐γ (IFN‐γ), tumor necrosis factor α (TNF‐α), Granzyme B and Perforin in αβ T cells (Figure S10, Supporting Information; Figure 5B),^[^ ^19^ ^]^ a feature unlikely to be the cause of tumorigenesis. As an integrin that mediates adhesion and tissue retention, CD103 expression usually distinguishes tissue‐resident lymphocytes from those in circulation.^[^ ^45^ ^]^ The infiltration of tissue‐resident T cells within tumors correlates with improved response to immune checkpoint blockades^[^ ^46^, ^47^ ^]^ and better clinical outcomes in cancer patients.^[^ ^48^, ^49^, ^50^ ^]^ The subsequent analysis of the subset of αβ T cells showed MED23 depletion resulted in a pan‐reduction of the major CD103^+^ αβ T cell (designated CD103^+^ T cell) subsets: CD4^+^ cells, CD8^+^ cells and CD4^−^ CD8^−^ cells (Figure S11, Supporting Information; Figure 5C–F). Overall, our results indicated that loss of MED23 upregulates the effector function of conventional T cells, and reduces the abundance of CD103^+^ T cells in the lung without impairing other immune cell populations. Likewise, declines of CD103^+^ T cells in *Med23^−/−^* mice were observed in nearly all the organs with spontaneous tumors (Figure 1F; Figure S12, Supporting Information), a pattern highly suggesting a conserved role for CD103^+^ T cells in controlling tumorigenesis.

To determine how MED23 mediates CD103^+^ T cell enrichment in lungs, we analyzed the levels of apoptosis and proliferation in CD103^+^ T cells from WT and *Med23* ^−/−^ lungs. As shown by the Annexin V and Propidium iodide (PI) staining, the WT and *Med23* ^−/−^ CD103^+^ T cells had similar levels of apoptosis (Figure 5G,H). In addition, the frequency of proliferating Ki67^+^ cells among CD103^+^ T cells in *Med23* ^−/−^ lungs demonstrated no obvious difference compared with the WT controls (Figure 5I,J). Previous studies have established T‐bet as a key transcription factor antagonizing the formation of CD103^+^ T_RM_ cells in the lung.^[^ ^51^, ^52^ ^]^ Thus, we examined T‐bet expression within αβ T cells and indeed observed that the frequency and cell number of T‐bet^+^ cells were significantly higher in *Med23* ^−/−^ αβ T cells compared with those in WT αβ T cells (Figure 5K–M). Moreover, we transplanted *Med23* ^−/−^ αβ T cells with T‐bet knock‐down into *Rag2* knockout (*Rag2* ^−/−^) hosts and observed increased generation of the major subset of CD103^+^ T cells, specifically CD103^+^ CD8^+^ T cells, in the lungs (Figure 5C; Figure S13A,B, Supporting Information). These results indicated that MED23 regulates CD103^+^ T cell generation by upregulating T‐bet expression.

Having found a defect in CD103^+^ T cell generation due to a lack of MED23 (Figure 5F), we sought to determine whether MED23 regulates the CD103^+^ T cell function. First, we analyzed the activating and inhibitory receptors on CD103^+^ T cells and found MED23 deficiency did not influence activating receptors (CD69, CD25, and CD44) and inhibitory receptors (PD‐1 and CTLA4) expression on CD103^+^ T cells (Figure S14A–D, Supporting Information).^[^ ^19^ ^]^ Considering that T cells secrete copious of cytokines in immune response to exert their functions,^[^ ^53^ ^]^ we examined cytokine production by CD103^+^ T cells in WT and *Med23* ^−/−^ lungs. WT and *Med23* ^−/−^ CD103^+^ T cells upregulated comparable amounts of IFN‐γ, interleukin 4 (IL‐4), interleukin 17 (IL‐17), TNF‐α, Granzym B and Perforin upon PMA and ionomycin stimulation (Figure S15A–G, Supporting Information), suggesting that MED23 is not a key regulator of CD103^+^ T cell effector function. Taken together, our results demonstrated that MED23 regulates the generation of lung CD103^+^ T cells without impacting their functional exertion.

### 2.5 CD103^+^ T Cells Prevent Tumorigenesis of AT2 Cell‐Derived Lung Adenocarcinoma

**Figure 6:**
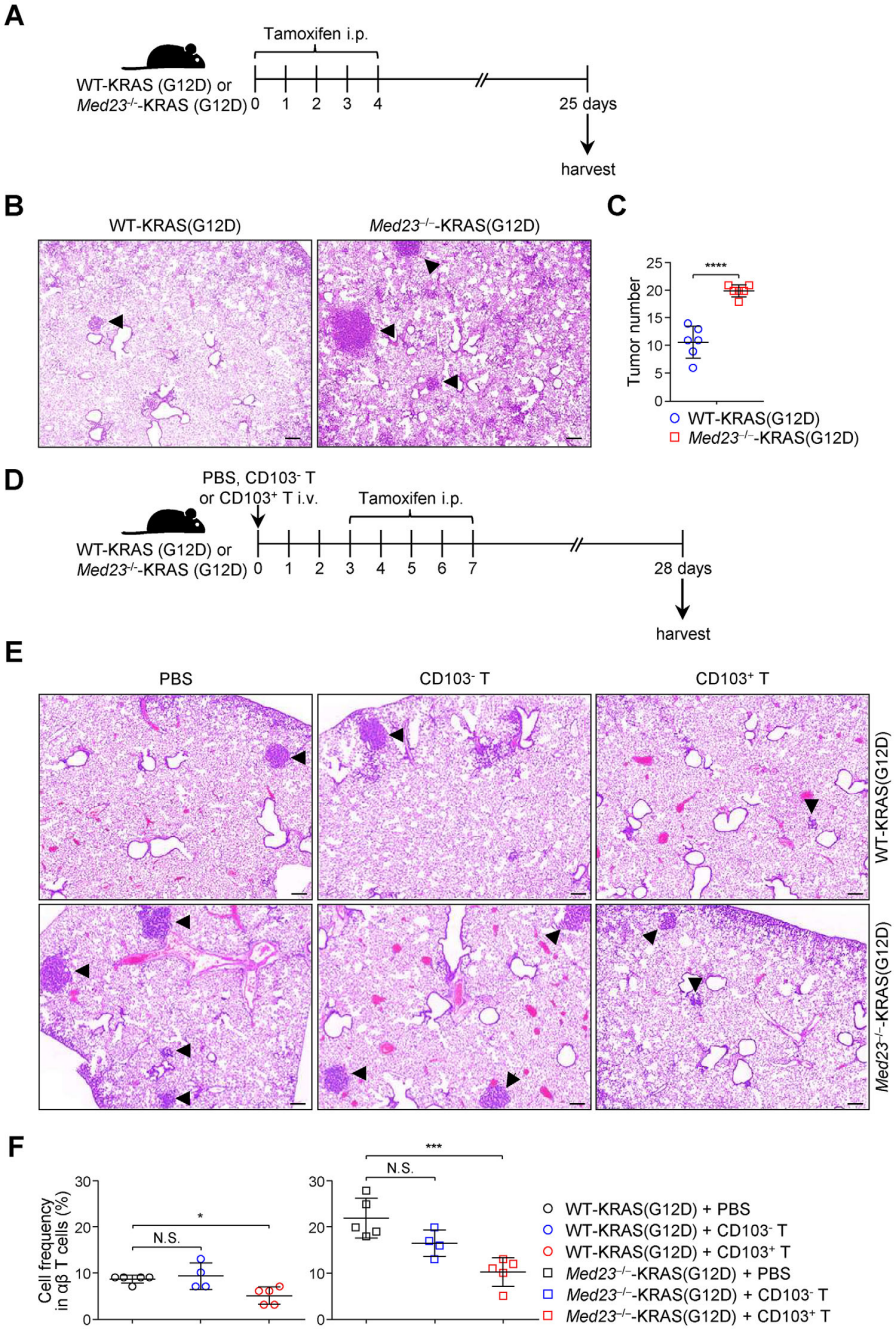
Lung CD103^+^ T cell transfer downregulates the tumor number of AT2 cell‐derived lung adenocarcinoma. A) Study design: WT‐KRAS (G12D) and *Med23* ^−/−^‐KRAS (G12D) mice received Tamoxifen for five consecutive days by i.p. injection at 4–5 weeks old. The lungs were collected 3 weeks after injection. B,C) Representative H&E sections B) and tumor numbers C) from WT‐KRAS (G12D) and *Med23* ^−/−^‐KRAS (G12D) lungs were analyzed (*n* = 6 mice). Arrowheads indicated lung tumors. Scale bar: 200 µm. D) Study design: WT‐KRAS (G12D) and *Med23* ^−/−^‐KRAS (G12D) mice received PBS, CD103^−^ T cell or CD103^+^ T cell treatment at 4–5 weeks old. On days 3 KRAS (G12D) activation in AT2 cells was began to induce. After tamoxifen injection 3 weeks, the lungs were harvested. E,F) Representative H&E‐stained sections E) and the tumor numbers F) were analyzed (PBS and CD103^+^ T: *n* = 5 mice; CD103^−^ T: *n* = 4 mice). Arrowheads indicated lung tumors. Scale bar: 200 µm. The data (C,F) are presented as the mean ± s.d. For all panels: ^*^ *p* < 0.05; ^***^ *p* < 0.001; ^****^ *p* < 0.0001 by Student's *t*‐test (C) or one‐way ANOVA with Tukey posthoc test F); N.S.: no significance. All data are representative of (B,E) or combined from (C,F) at least three independent experiments.

To further elucidate the function of CD103^+^ T cell in preventing oxidative damaged cell‐derived tumorigenesis, we bred WT and *Med23* ^−/−^ mice with *Sftpc‐DreER*; *K‐ras* ^Rox–Stop–Rox–G12D/+^ mice (designated *Med23* ^−/−^‐KRAS (G12D) mice), which express the mutant KRAS (G12D) in AT2 cells upon tamoxifen administration and develop ROS‐dependent lung adenocarcinoma at 9 weeks old (**Figure** **6**A,B).^[^ ^54^ ^]^ Histology analysis revealed that *Med23* ^−/−^ mice developed more tumors after the induction of KRAS (G12D) (Figure 6B,C). We next transferred purified CD103^+^ T cells from the lung into *Rag2* ^−/−^ mice and investigated CD103^+^ T cell distribution in the spleen, lung, liver, and kidney. As shown in Figure S16 (Supporting Information), the transferred CD103^+^ T cells were primarily detected in the lung, highlighting the tissue‐specificity of these cells. To further confirm the role of CD103^+^ T cells during lung adenocarcinoma initiation, we adoptively transferred purified CD103^−^ T cells or CD103^+^ T cells into WT‐KRAS (G12D) and *Med23* ^−/−^‐KRAS (G12D) mice and induced the KRAS (G12D) expression after that (Figure 6D). Notably, both WT‐KRAS (G12D) mice and *Med23* ^−/−^‐KRAS (G12D) mice receiving CD103^+^ T cells exhibited a significant decrease in tumor number compared with the mice that received CD103^−^ T cells (Figure 6E,F). These data indicated that CD103^+^ T cells exhibit the distinct ability to prevent the tumorigenesis of lung adenocarcinoma arising from oxidative stress‐bearing AT2 cells.

### 2.6 CD103^+^ T Cells Eliminate Damaged AT2 Cells Bearing Oxidative Stress

**Figure 7:**
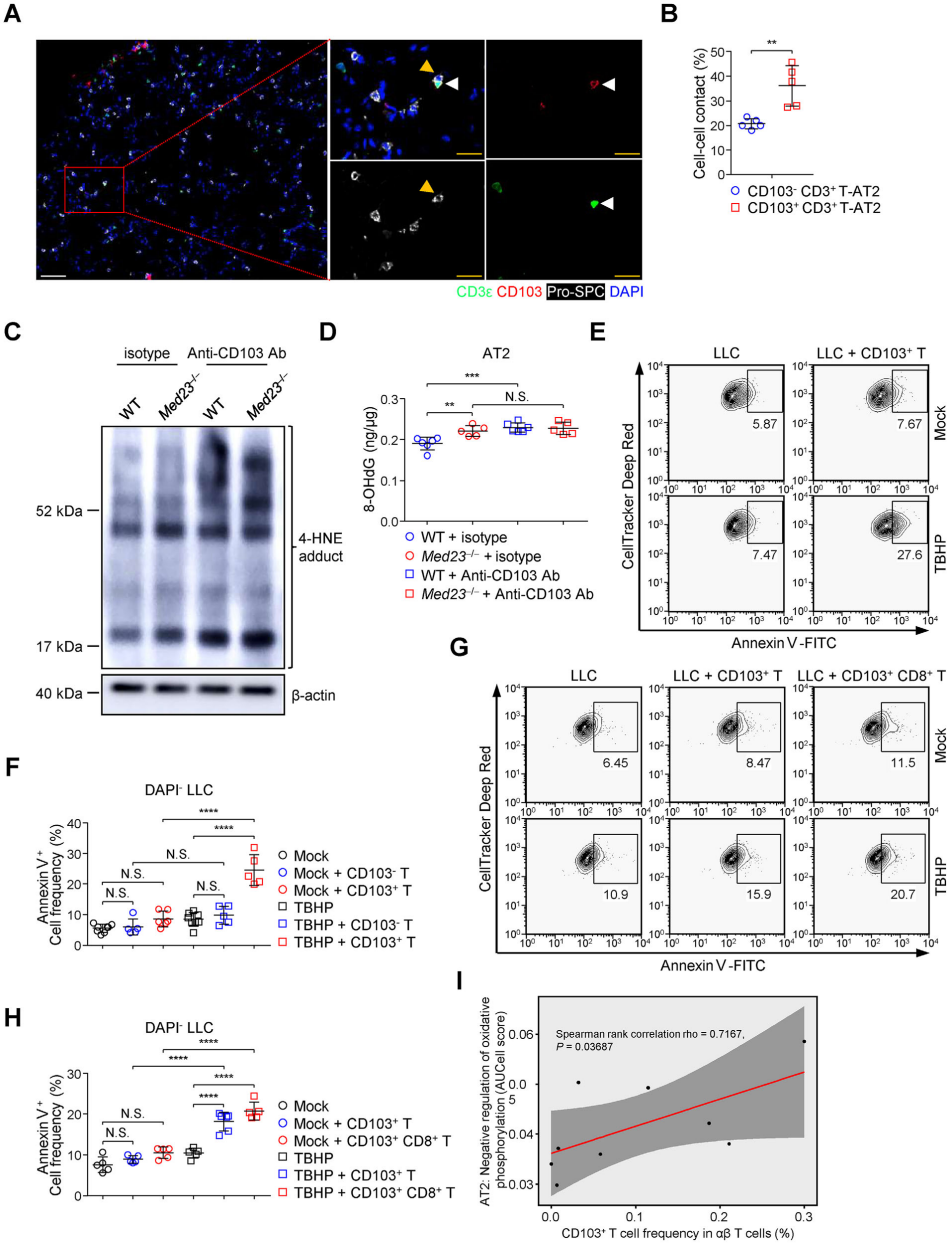
CD103^+^ T cells decrease the accumulation of damaged AT2 cells bearing oxidative stress in the lungs. A) Representative immunofluorescence staining for CD3ε, CD103, and pro‐SPC in lungs. Arrowhead: white, CD103^+^ CD3^+^ T cell; orange, AT2 cell. Scale bar: white, 50 µm; orange, 10 µm. B) The percentage of CD103^−^ or CD103^+^ CD3^+^ T cell‐AT2 cell contacts in lungs (*n* = 5 mice). Cell‐cell contact: nucleus‐to‐nucleus distance < 12 µm (the sum of cell radius of CD103^−^ or CD103^+^ CD3^+^ T cell and AT2 cell). C,D) WT and *Med23* ^−/−^ mice received isotype or CD103 antibody by i.p. injection on days 0, 7, 14 and 21. On days 28, WT and *Med23* ^−/−^ AT2 cells were isolated and their 4‐HNE C) and 8‐OHdG expression D) were assessed (WT: *n* = 6 mice; *Med23* ^−/−^: *n* = 5 mice). E,F) After treated with mock or TBHP (200 µm), LLC cells either cultured alone or co‐cultured with CD103^+^ T cells for 6 h. Representative flow cytometry analysis E) of Annexin V expression in DAPI^−^ LLC cells were displayed. The percent F) of Annexin V^+^ cells in DAPI^−^ LLC cells in the presence of the indicated conditions were analyzed (Mock and TBHP: *n* = 8 biologically repeated samples; Mock + CD103^−^ T, TBHP + CD103^−^ T and TBHP + CD103^+^ T: *n* = 5 biologically repeated samples; Mock + CD103^+^ T: *n* = 6 biologically repeated samples). G,H) After treated with mock or TBHP (100 µm), LLC cells either cultured alone or co‐cultured with CD103^+^ T cells or CD103^+^ CD8^+^ T cells for 6 h. Representative flow cytometry analysis G) of Annexin V expression in DAPI^−^ LLC cells were displayed. The percent H) of Annexin V^+^ cells in DAPI^−^ LLC cells in the presence of the indicated conditions were analyzed (Mock, Mock + CD103^+^ T, Mock + CD103^+^ CD8^+^ T, TBHP and TBHP + CD103^+^ CD8^+^ T: *n* = 5 biologically repeated samples; TBHP + CD103^+^ T: *n* = 6 biologically repeated samples). I) Scatter plots showing that in human lungs, the percentage of CD103^+^ T cells in αβ T cells and the AUCell score of gene expression related to negative regulation of oxidative phosphorylation from AT2 cells (*n* = 9 human lung samples). The red line represented the regression line of CD103^+^ T cell frequency and AUCell score and the dark grey region represented the 95% CI of the regression line. The data (B, D, F, H) are presented as the mean ± s.d. For all panels: ^**^ *p* < 0.01; ^***^ *p* < 0.001; ^****^ *p* < 0.0001 by Student's *t*‐test (B) or one‐way ANOVA with Tukey posthoc test (D, F, H); N.S.: no significance. The data are representative of (A, C, E, G) or combined from (B, D, F, H, I) at least three independent experiments.

Having observed a connection between CD103^+^ T cells and AT2 cells, we performed immunofluorescence staining to look at the location of the CD103^+^ T cells in lungs from adult WT mice and found that T cells expressing CD103 exhibited a closer contact with AT2 cells as compared to the CD103^−^ ones (**Figure** **7**A,B), suggesting a potential immuno‐regulating role of the CD103^+^ T cells toward AT2 cells. To further elucidate the functional role of CD103^+^ T cells, we first successfully depleted CD103^+^ T cells in the lungs with no significant influence on other CD103^+^ cell cellularity by using the neutralizing antibody against CD103 and further analyzed the oxidative stress in AT2 cells in WT and *Med23* ^−/−^ mice (Figure S17, Supporting Information). Interestingly, CD103^+^ T cell depletion not only facilitated 4‐HNE accumulation in both WT and *Med23* ^−/−^ AT2 cells, but also increased 8‐OHdG accumulation in WT AT2 cells (Figure 7C,D). To determinate whether CD103^+^ T cells display cytotoxicity against cells under oxidative stress, we set up a co‐culture system in vitro in which purified CD103^+^ T cells were co‐incubated with target cells bearing different levels of oxidative stress. By tert‐butyl hydrogen peroxide (TBHP) treatment, we induced a high‐level of oxidative stress in Lewis Lung Carcinoma (LLC) cells without affecting cell viability (Figure 7E; Figure S18, Supporting Information). After co‐culture with CD103^+^ T cells, LLC cells pre‐treated with TBHP exhibited significantly higher percentages of apoptosis, whereas the ones that co‐cultured with CD103^−^ T cells stayed at the same levels of apoptosis as the controls (Figure 7E,F; Figure S19, Supporting Information). To further uncover the mechanism by which CD103^+^ T cells induce oxidative stressed cell apoptosis, we investigated which subset of CD103^+^ T cells elicit this action. As shown in Figure 7G,H (Figure S18, Supporting Information), CD103^+^ CD8^+^ T cells, the major portion of lung CD103^+^ T cells (Figure 5C), displayed an increased tendency of cytotoxicity toward oxidative stressed cells compared with CD103^+^ T cells, implying their substantial role in elimination of oxidative stressed cells. These results suggested CD103^+^ CD8^+^ T cells might be the major functional subset within the CD103^+^ T cells scavenging oxidative stressed cells. When we analyzed the single‐cell sequencing data of human lung samples,^[^ ^55^ ^]^ we observed a significant positive correlation between the percentage of CD103^+^ T cells among αβ T cells and the AT2 cell gene expression related to negative regulation of oxidative phosphorylation which implies low‐level of oxidative stress (Figure 7I).^[^ ^56^ ^]^ Together, these results proved CD103^+^ T cells act as a key cell type that surveils somatic cells under oxidative stress, which prevents malignancy arising from oxidative DNA damages.

### 2.7 CD103^+^ T Cells in the Lung are Declining with Age

**Figure 8:**
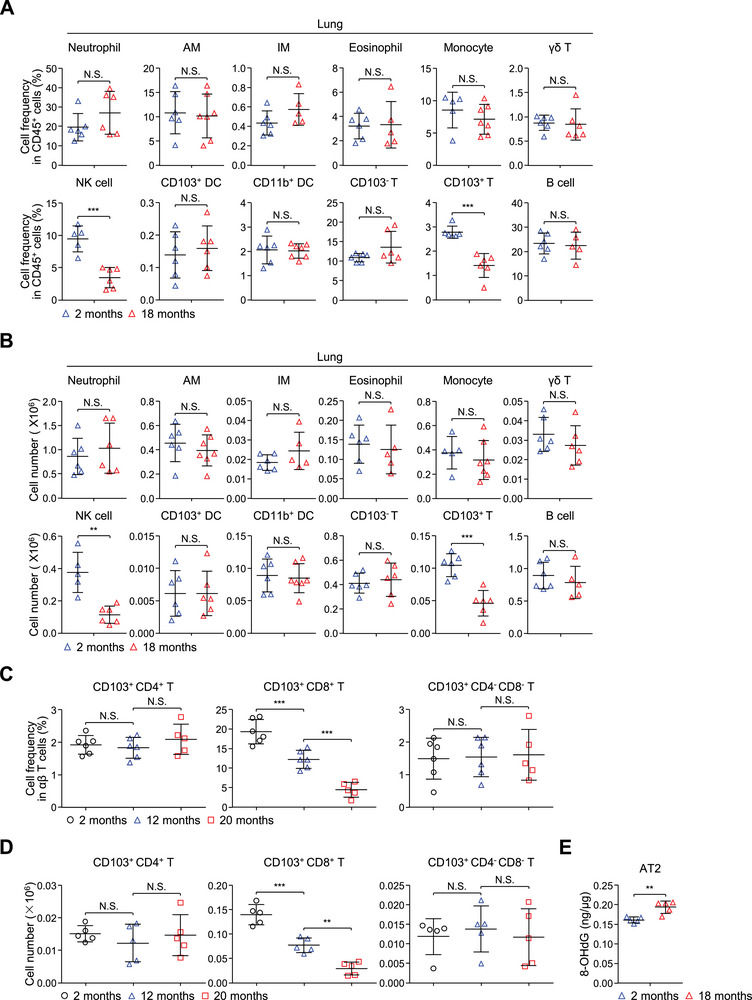
A decrease of CD103^+^ T cells in the lungs is accompanied by increased oxidative damage in AT2 cells from aged mice. A,B) The percentage A) and absolute number B) of neutrophils, alveolar macrophages (AM), interstitial macrophages (IM), eosinophils, monocytes, γδ T cells, NK cells, CD103^+^ DCs, CD11b^+^ DCs, CD103^−^ T cells, CD103^+^ T cells and B cells in lungs from 2‐month‐old and 18‐month‐old mice (Neutrophil, γδ T, CD103^+^ DC, CD103^−^ T and CD103^+^ T: *n* = 6 mice; AM and CD11b^+^ DC: 2 months, *n* = 6 mice, 18 months, *n* = 7 mice; IM, Eosinophil and B cell: 2 months, *n* = 6 mice, 18 months, *n* = 5 mice; Monocyte: 2 months, *n* = 5 mice, 18 months, *n* = 7 mice; NK cell: 2 months, *n* = 5 mice, 18 months, *n* = 6 mice). C,D) The percentage C) and cell number D) of CD103^+^ CD4^+^ T cells, CD103^+^ CD8^+^ T cells, and CD103^+^ CD4^−^ CD8^−^ T cells in lungs from 2, 12, 20‐month‐old C57BL/6 mice (percentage: 2 months and 12 months, *n* = 6 mice, 20 months, *n* = 5 mice; cell number: *n* = 5 mice). E) Elisa of 8‐OHdG expression in isolated AT2 cells of 2‐month‐old and 18‐month‐old WT mice (*n* = 5 mice). The data (A‐E) are presented as the mean ± s.d. For all panels: ^**^ *p* < 0.01; ^***^ *p* < 0.001 by Student's *t*‐test (A, B, E) or one‐way ANOVA with Tukey post‐hoc test (C, D); N.S.: no significance. All data are combined from (A‐E) at least three independent experiments.

Lung cancer is the most frequent cause of cancer‐related death worldwide, with aging as a prominent risk factor.^[^ ^22^, ^57^ ^]^ To unravel the association of CD103^+^ T cell cellularity with age, we used flow cytometry to profile aging‐associated immune changes in the lung from C57BL/6 mice (Figure S20A–C, Supporting Information). As shown in **Figure** **8**A,B, the abundance of the majority of myeloid and lymphoid populations in the lung was comparable between the young (2 months old) and the aged (18 months old) mice, whereas significant decreases in NK cells and CD103^+^ T cells were detected in the aged group. Notably, the percentage and absolute number of CD103^+^ CD8^+^ T cells obviously declined with age compared with the other subsets of CD103^+^ T cells (Figure 8C,D). Likewise, elevated 8‐OHdG in AT2 cells was observed in aged mice (Figure 8E). These results indicated the association between CD103^+^ T cells and oxidative stressed epithelial cells co‐exists in the aged lung. Together, the above findings suggest the aging‐associated decline of CD103^+^ T cells in the lung might be a key defect contributing to the high incidence of lung cancer in the aged population.

## 3 Discussion

Using a mouse model with *Med23* deletion in T cells, we uncover a strong association between the decline of CD103^+^ T cells and the tumorigenesis of AT2‐originated adenocarcinoma in the lung. In‐depth analysis reveals that CD103^+^ T cell reduction leads to the accumulation of damaged AT2 cells under oxidative stress which largely account for the tumorigenesis of lung adenocarcinoma. Mechanistically, we show that loss of MED23 upregulated T‐bet, a transcription factor antagonizing the formation of CD103^+^ T cells and these CD103^+^ T cells eliminate somatic cells under oxidative stress in vivo and in vitro. These findings demonstrate that CD103^+^ T cells within tissue play a direct role in surveilling somatic cells with oxidative stress to prevent tumorigenesis and reveal the importance of MED23 in controlling CD103^+^ T cell generation. Moreover, we identified the decline of CD103^+^ T cells as a significant aging‐associated alteration within the lung infiltrating immune cells, which might contribute to the accumulation of AT2 cells under oxidative stress and lung cancer.

Emerging evidence reveals that aging induces a broad range of changes to the immune cells which reprogram the immune‐surveilling system thereby contributing to the rise of cancer incidence.^[^ ^58^, ^59^, ^60^ ^]^ As the central player in adaptive immunity, T cells in aged individuals acquire several aging‐associated features which manifest as immunodeficiency and inflammaging at the same time.^[^ ^61^ ^]^ Although functional deterioration of aged T cells is recognized of critical importance during cancer initiation and progression,^[^ ^60^, ^62^, ^63^ ^]^ the nexus between T cell dysregulation and tumorigenesis in the aged population remains poorly defined. A recent study demonstrated that aging specifically and dramatically decreases the generation of CD8^+^ T_RM_ cells in various tissues, which compromised the antitumor immunity of aged CD8^+^ T cells.^[^ ^64^ ^]^ In this study, we also observed the aging‐associated decline of CD103^+^ CD8^+^ T cells in the lung (Figure 8B–D). In addition, we found CD103^+^ CD8^+^ T cells display robust cytotoxicity against cells under oxidative stress (Figure 7G,H). Our study implies that the decline of CD103^+^ T cells in the aged lung can compromise the immune surveillance against cells under oxidative stress and may subsequently contribute to the ROS‐dependent tumorigenesis of adenocarcinomas. Taken together, these studies underscore the critical role of T_RM_ cells, especially CD8^+^ T_RM_ cells in controlling tumor initiation and progression, and demonstrate the dysregulated immune surveillance within tissues as a key mechanism underlying the high prevalence of tumors in the aged population. Given the decline of CD103^+^ CD8^+^ T cells in the lung appears to be a shared feature between *Med23* ^−/−^ and aged animals, whether MED23 plays a physiological role during T cell aging deserves future exploration. In addition, aging‐associated decline of CD8^+^ T_RM_ cells was also observed in human lung samples,^[^ ^65^ ^]^ implying targeting the dysregulation of T_RM_ cells within tissues might be a novel strategy to improve cancer treatment.

Lung cancers are one of the most common cancers and the leading cause of cancer‐related death of both males and females.^[^ ^57^, ^66^ ^]^ Lung adenocarcinoma is the one representing ≈40% and the most common type of lung cancer.^[^ ^67^, ^68^ ^]^ The high incidence of lung cancers in the aged population has long been observed,^[^ ^22^ ^]^ but the underlying mechanisms remain unclear. We show that the AT2 cells under oxidative stress accumulate with age due to compromised T cell surveillance, and give rise to lung adenocarcinomas (Figures 6, 7, and 8B–E).^[^ ^54^ ^]^ Similar to ER stress that serves as a target of NK cells,^[^ ^69^ ^]^ our study for the first time, reveals the oxidative stress signals to the regional immune system to purge the pre‐neoplastic cells. These findings together emphasize the complex roles of the aging‐associated cellular events during tumor initiation and progression. It's noteworthy that although oxidatively damaged AT2 cells appear to be the major origin of lung adenocarcinoma in our model due to the decline of CD103^+^ T cells, other somatic cells in lungs such as AT1 cells under oxidative stress might also be surveilled by these resident T cells as well.

An association between the abundance of tumor T_RM_ cells and improved clinical outcome has been observed in cancer patients,^[^ ^70^ ^]^ and functional studies revealed that tumor T_RM_ cells are potent “killers” with direct cytotoxicity against tumors.^[^ ^71^, ^72^ ^]^ Nevertheless, whether, and how tissue‐resident T cells patrol and respond to transformed cells in situ prior to tumor formation remain unclear. Here we find CD103^+^ T cells directly induce cell death to target cells with augmented oxidative stress and CD103^+^ T cell transfer prevents tumorigenesis of ROS‐dependent lung adenocarcinoma (Figures 6E,F and 7E–H).^[^ ^54^ ^]^ These results suggest that CD103^+^ T cells directly eradicate pre‐neoplastic cells via sensing stress‐related molecules or antigens with oxidation‐related modifications. Compared with conventional T cells which recognize neoantigens, non‐self‐antigens (e.g. viral antigens) or overexpressed self‐antigens with the help from antigen‐presenting cells in tumor immunity, CD103^+^ T cells display a direct response to cellular stress related to transformation and tumorigenesis. Moreover, the reduction of CD103^+^ T cells in the lung, the liver and the small intestine is accompanied by spontaneous epithelial cell‐derived tumors in these tissues of *Med23* ^−/−^ mice (Figure S12, Supporting Information; Figure 1F), implying the importance of CD103^+^ T cells in surveilling epithelial cell‐derived tumor.

MED23, a key component of the tail modules of the mediator complex, exerts distinct functions in various biological processes, such as hematopoietic stem cell differentiation, carcinogenesis, and angiogenesis.^[^ ^73^, ^74^, ^75^ ^]^ It's noteworthy that our previous research has displayed that *Med23* deletion significantly increases the effector function of conventional T cells, thereby repressing tumor development in PyMT model, in which the expression of middle T oncogene in mammary epithelium is prior to the full establishment of T_RM_ cells. In this study, we found *Med23* deletion in T cell compartment reduces CD103^+^ T cells, which give rise to an accumulation of oxidative stressed AT2 epithelial cells which appear to be a key origin of adenocarcinoma during aging (Figures 5E,F and 7C,D). These results emphasize the specific function of MED23 in facilitating the development and function of different T‐cell subsets. Moreover, our results suggest that immune surveillance might rely on different cell types within different organs.

## 4 Experimental Section

### Mice

*Med23* ^fl/fl^ *Cd4*‐*Cre* mice were generated as previously described.^[^ ^20^ ^]^ *Med23* ^fl/fl^ mice were maintained and backcrossed to a B6 background for at least 10 generations. *Cd4*‐*Cre* transgenic mice were obtained from Professor Z. Hua (Nanjing University). *Rag2* ^−/−^ mice were obtained from the Institute of Development Biology and Molecular Medicine (Fudan University) and maintained on a B6 background. *Sftpc‐DreER* mice had been obtained from Professor B. Zhou (Center for Excellence in Molecular Cell Science, Chinese Academy of Science).^[^ ^24^ ^]^ *K‐ras^Rox–Stop–Rox–G12D/+^* mice were obtained from Professor H. Ji (Center for Excellence in Molecular Cell Science, Chinese Academy of Science). C57BL/6 mice were purchased from GemPharmatech Co., Ltd. All mice were maintained in Specific Pathogen Free (SPF) conditions and genotyped by PCR before experimentation. Both male and female mice 2–6 months, 12 months, and 18‐20 months of age were used. Mice were randomly assigned to experimental groups and processed to ensure the reliability of the conclusions. It was not blind to group assignments during the experiments and results evaluation. All animal experiments were conducted according to the guidelines of the Institutional Animal Care and Use Committee (IACUC) at the Center for Excellence in Molecular Cell Science, Shanghai Institutes of Biological Sciences, Chinese Academy of Science (approval number, IACUC‐IBCB0013). The mouse information used in this study is listed in Table S2 (Supporting Information). The sequences of primers used for genotyping are listed in Table S3 (Supporting Information).

### Cell Culture

The Plat‐E cell line was obtained from Professor G. Pei (Center for Excellence in Molecular Cell Science, Chinese Academy of Science) and was originally purchased from Cell Biolabs, Inc. The LLC cell line was obtained from the Cell Bank of the Chinese Academy of Sciences. The cells were cultured in Dulbecco's modified Eagle's medium (DMEM) supplemented with FBS (10%, Sunrise) and penicillin/streptomycin (1%, Gibco) in a humidified incubator at 37 °C, 5% CO_2_. All cell lines used were tested and shown to be mycoplasma‐free.

### Tissue Preparation and Cell Isolation

Thymocytes were prepared by grinding and filtering through a nylon screen. Splenocytes were obtained by squeezing and lysing red blood cells before filtration.^[^ ^20^ ^]^ The lungs, livers, and kidneys were perfused with cold PBS to remove the blood cells before collection. Then, the lungs were cut into pieces and incubated with shaking (900 rpm) at 37 °C for 40 min in RPMI medium (3 mL, Gibco) containing FBS (10%, Sunrise), β‐mercaptoethanol (50 µm) and collagenase I (70 U ml^−1^, Gibco). The livers were minced and filtered through a cell strainer (40 µm, BD Biosciences). The kidneys were cut into pieces and incubated with rotating at 37 °C for 90 min in RPMI medium (Gibco) containing FBS (5%, Sunrise), DNase I (0.2 µg mL^−1^, Roche), HEPES (10 mm), β‐mercaptoethanol (50 µm) and collagenase IV (0.2 mg mL^−1^, Sigma). The small intestine was dissected after Peyer's patches movement, flushed with cold PBS, opened longitudinally, and cut into small fragments roughly 2–4 mm in length. Intestine fragments were washed twice with cold PBS and then incubated with EDTA‐PBS (20 mm) at 37 °C for 30 min. Leukocytes were isolated from the digested tissues, liver cell suspensions, and supernatant of intestine fragments by density fractionation using discontinuous 40–70% (vol/vol) Percoll (GE Healthcare) gradients.

### FACS Analysis and Cell Sorting

For cell surface staining, preincubated cell suspensions in Fc Block (BD Biosciences) and then stained for 40 min at 4 °C with the indicated antibodies. For the analysis of cytokine secretion in CD103^+^ T cells, lung leukocytes were stimulated in RPMI medium (Gibco) containing FBS (10%, Sunrise), β‐mercaptoethanol (50 µm), PMA (50 ng mL^−1^, Merck), ionomycin (1 ug mL^−1^, Merck) and brefeldin A (1000×, eBioscience) for 3.5 h. Then the cells were harvested, and the intracellular staining for cytokines was performed after 10 min of fixation with paraformaldehyde solution (4%) in PBS at room temperature and 5 min of permeabilization in permeabilization buffer (eBioscience) at 4 °C. Intracellular staining for T‐bet and Ki67 was performed using a Foxp3 staining kit (eBioscience) according to the manufacturer's protocol. ROS production of neutrophils and macrophages in lungs was measured by CellROX™ Deep Red (Invitrogen) according to the manufacturer's protocol. Cell fluorescence was performed on a four‐laser BD LSRFortessa and the acquired data were analyzed with FlowJo software (TreeStar, Inc., Olten, Switzerland). Cell sorting was performed with a BD FACSAria III after surface staining. The sorted cell purity was greater than 95%. Fluorescently conjugated proteins or antibodies used for cell‐surface staining and intracellular staining are listed in Table S4 (Supporting Information).

### Lung Adenocarcinoma Model

WT‐KRAS (G12D) and *Med23* ^−/−^‐KRAS (G12D) mice received Tamoxifen (200 µL, 5 mg mL^−1^, ABCone) for five consecutive days by i.p. injection at 4–5 weeks old. The lungs were collected for H&E staining and analysis of tumor numbers 3 weeks after injection. For the adoptive cell transfer experiment, WT‐KRAS (G12D) and *Med23* ^−/−^‐KRAS (G12D) mice received 1 × 10^5^ sorted lung CD103^+^ T cells (CD103^+^ TCRβ^+^) or CD103^−^ T cells (CD103^−^ TCRβ^+^) by i.v. injection 3 days before mice were treated with Tamoxifen.

### Quantitative Real‐Time PCR Analysis

Total RNA from lung tissues was extracted by TRIzol (Invitrogen). Reverse‐transcribed using the SuperScript III First‐Strand Synthesis System (Invitrogen). SYBR Green QPCR Master Mix (Toyobo) was used for gene amplification and detection. The mRNA levels of the indicated genes were normalized to β‐actin using real‐time PCR (LightCycler 96; Roche).^[^ ^76^ ^]^ The sequences of qPCR primers are listed in the Table S3 (Supporting Information).

### Isolation of Mouse Alveolar Epithelial Type II cells

Lung tissues were dissociated with a dispase solution as previously described.^[^ ^77^ ^]^ Briefly, after the lungs were perfused with cold PBS through the right ventricle to remove the blood cells, 1 mL dispase buffer containing dispase (50 U mL^−1^, Sigma) and FBS (2%, Sunrise) was instilled into the lungs through the trachea, followed by the instillation of 1% low‐melt agarose (0.6 mL, Sigma). Then the lungs were covered with crushed ice for 2 min for the agarose to solidify. After extraction, the lungs were cut into individual lobes and dropped into a 50 mL conical tube containing of dispase buffer followed by rotating incubation (300 rpm) for 45 min at 37 °C. The lobes of lung were gently teased away from the large airways by using sharp tweezers. The cell suspensions were filtered through a cell strainer (40 µm, BD Biosciences) and centrifuged at 300 g for 10 min at 4 °C. The cell pellets were resuspended in DMEM medium containing FBS (10%, Sunrise) and DNase I (50 U mL^−1^, Roche) for further magnetic enrichment of AT2 cells using Dynabeads Biotin Binder (Invitrogen) according to the manufacturer's protocol. The biotinylated antibodies used were as follows: anti‐CD45 (30‐F11, Biolegend), anti‐CD31 (MEC13.3, Biolegend), anti‐Ter119 (TER‐119, Biolegend), anti‐integrin β4 (346‐11A, Biolegend). The purity of AT2 cell negative selection was greater than 90% by immunofluorescence to assess the expression of pro‐SPC. Alternatively, the cell pellets were lysed red blood cells and then stained for sorting. Live AT2 cells were sorted with a CD45^−^ CD326^+^ CD31^−^ CD104^−^ gating strategy.

### Antioxidant NAC Administration

Mock or NAC (40 mm, Sigma) was supplemented in the drinking water of mice and freshly prepared every 7 days.^[^ ^78^ ^]^ For 4HNE and 8‐OHdG assay of AT2 cells, 2‐month‐old mice were administered by mock or NAC for 4 months. For the analysis of spontaneous lung adenocarcinoma, 12‐month‐old mice were administered mock or NAC for 6 months.

### T‐bet Knock‐Down in T Cells

To clone T‐bet‐shRNA expression plasmid, the oligo pairs (5′‐TGCTGTTGACAGTGAGCGCCCATTTTCAGTAAAAAGGAATAGTGAAGCCACAG‐3′; 5′‐GAAGCCACAGATGTATTCCTTTTTACTGAAAATGGGTGCCTACTGCCTCGGA‐3′) were amplified using XhoI and EcoRI primers through PCR, and the resulting products were subsequently cloned into the pMLP vector. The pMLP vector was obtained from Professor H. Jiang (Center for Excellence in Molecular Cell Science, Chinese Academy of Science). The constructed plasmids were transfected into Plat‐E cells using Lipo8000 (Beyotime). The viral supernatants were collected at 48 h and 72 h post‐transfection. RetroNectin (20 µg mL^−1^, Takara) was diluted in PBS for the coating of non‐treated 24‐well plates overnight at 4 °C. The plates were then blocked with PBS containing FBS (2%, Sunrise) at room temperature for 1 h and the viral supernatants were added and centrifuged at 30 °C at 2000 rpm for 3.5 h. The isolated spleen‐derived naïve T cells were pre‐activated via anti‐CD3ε (1.5 µg mL^−1^, BD Biosciences, 553238) and anti‐CD28 (1.5 µg mL^−1^, BD Biosciences, 553295) for 24 h. After removing the viral supernatants, the activated T cells were added and centrifuged at 30 °C at 800 rpm for 30 min, followed by incubation at 37 °C for 3 h. Subsequently, the 1 × 10^6^ transduced T cells were adoptively transferred into *Rag2* ^−/−^ mice by retro‐orbital injection. Flow cytometric analysis of CD103 expression in lung T cells on days 7.

### In Vivo Depletion of CD103^+^ Cells

To deplete CD103^+^ cells in vivo, mice were given injected intraperitoneally with anti‐CD103 mAb (200 µg, M290, BioXCell) on days 0, 7, 14, and 21. As a control, the same dose of IgG2a isotype (BioXCell) was used in the same manner. Lungs were harvested and AT2 cells were further isolated for 4HNE and 8‐OHdG assay on days 28.

### Histological Procedures

Sections cut from paraformaldehyde (4%)‐fixed, paraffin‐embedded blocks of mice tissues from lungs were used for H&E staining or immunofluorescence, and from thymus, liver, small intestine, and blood vessels were used for H&E staining. To analyze the phenotype of lung tumors from aged mice, the paraffin sections of lungs were stained using a Two color mIHC Fluorescence kit (Recordbio Biological Technology) according to the manufacturer's instructions. To assess the distance between CD103^+^ T cells and AT2 cells and the AT2 cell proliferation, lungs were perfused with cold PBS through the right ventricle to remove blood cells, and cold paraformaldehyde (0.8 mL, 4%) was instilled into the lungs through the trachea. Then the lungs were subjected to continued fixing in paraformaldehyde (4%) for 1 h at 4 °C. After washing with PBS three times, the lungs were dehydrated in sucrose (30%) overnight and embedded in the OCT (Sakura). The lung sections (8 µm) were blocked by PBS containing donkey serum (5%, Jackson) and Triton X‐100 (0.3%, Sigma) for 30 min at room temperature and then incubated with specific antibodies overnight at 4°C. After washing with PBS three times, the lung sections were incubated with corresponding secondary antibodies at room temperature for 1 h. Finally, the lung sections were incubated for 10 min with DAPI (abcam). For labeling AT2 cells with EdU in vivo, EdU (50 ug g^−1^) was intraperitoneally injected into mice on days 0, 7, 14, and 21. Lungs were collected on day 28 and the lung sections were performed using the EdU Imaging Kit (Invitrogen). The primary antibodies including rabbit anti‐pro‐SPC (Sigma, AB3786), rabbit anti‐CC10 (abcam, ab213203), rabbit anti‐Keratin 5 (BioLegend, 905501), Alexa Fluor 594‐conjugated rat anti‐CD3 (BioLegend, 100240) and FITC‐conjugated hamster anti‐CD103 (BioLegend, 121419). The secondary antibodies include Alexa Fluor 568‐conjugated goat anti‐rabbit IgG (Invitrogen, A‐11036) and Alexa Fluor 647‐conjugated donkey anti‐rabbit IgG (Invitrogen, A‐31573). Images were obtained in Olympus SpinSR and Pannoramic DESK.

### Western Blotting

Isolated AT2 cells were washed twice with cold PBS and then lysed in SDS loading buffer containing β‐mercaptoethanol (15 mm). Proteins were separated by SDS–PAGE and transferred onto PVDF membranes (Sigma, GE10600023). The PVDF membranes were blocked with TBST containing skim milk (5%) and further incubated with primary antibodies overnight at 4 °C. After washing with TBST, the PVDF membranes were incubated with HRP‐conjugated secondary antibodies. The primary antibodies include mouse anti‐4HNE (abcam, ab48506) and rabbit anti‐β‐Actin (ABclonal, AC026). The secondary antibodies including HRP‐conjugated anti‐mouse lgGκ (Santa, sc‐516102) and anti‐rabbit lgG (Santa, sc‐2357).

### Analysis of the DNA Damage Types

The genomic DNAs of lungs and AT2 cells were isolated using the Genomic DNA Purification Kit (Promega) and were digested with Nuclease P1 (NEB) and CIAP (Takara). Then the nucleotides were centrifuged at 5000 g for 40 min at room temperature using a Nanosep centrifugal device (Pall Corporation) to remove the proteins. For the HPLC‐MS analysis, the nucleosides of lungs were quantified using a 6495B Triple quadrupole mass spectrometry equipped with an ESI probe and 1260 HPLC system (Agilent U.S.A.) Accucore aQ column (100 mm × 2.1 mm id, 2.6 µm particle size, Thermo Fisher, Scientific) coupled with a corresponding guard column (10 mm × 2.1 mm id, 2.6 µm particle size, Thermo Fisher, Scientific) was employed at 25 °C and autosampler was set to 4 °C. Analysis was conducted based on Su's method with modification.^[^ ^79^ ^]^ Briefly, a binary gradient of solvent A of water (100%) containing formic acid (0.1%) and solvent B of acetonitrile (100%) containing formic acid (0.1%) was used for gradient elution: 0–2 min, A (100%); 2–10 min, A (100%) to A (95%); 10–12 min, A (95%) to A (20%); 12–15 min, A (20%) to A (100%); a flow rate (0.3 mL min^−1^) was used. The total run time was 15 min. The column was equilibrated for 5 min at A (100%) between injections. The MS was operated in positive mode. The following parameters were optimized for nucleosides analysis: drying gas temperature (250 °C), drying gas flow (14 L min^−1^), sheath gas temperature (300 °C), sheath gas flow (11 L min^−1^), nebulizer pressure (20 psi), capillary voltage (4000 V), fragmentor voltage (380 V), RF voltage amplitudes of high‐pressure and low‐pressure ion funnels are 150 and 60 V respectively. MRM transitions of nucleosides are listed in Table S5 (Supporting Information). For the Elisa of 8‐OHdG, the nucleosides of AT2 cells were used to measure 8‐OHdG by an 8‐hydroxy 2 deoxyguanosine ELISA Kit (abcam) according to the manufacturer's protocol.

### Cytotoxic Assay of T Cells

After being labeled with 5 µm CellTracker Deep Red dye (Invitrogen), LLC cells were seeded into 96‐well plates (1 × 10^4^ cells per well) and treated with Mock or indicated dose of TBHP (Sigma) for 2 h at 37 °C. The pre‐treated LLC cells were cultured alone or co‐cultured with sorted 1 × 10^5^ CD103^+^ T cells (CD103^+^ TCRβ^+^), CD103^+^ CD8^+^ T cells (CD103^+^ CD8^+^ TCRβ^+^) or CD103^−^ T cells (CD103^−^ TCRβ^+^) for 6 h at 37 °C. The apoptosis of LLC cells was measured by Annexin V and DAPI staining.

### CD103^+^ T Cell Enrichment Analysis

*Rag2* ^−/−^ mice received 1 × 10^5^ sorted lung‐derived CD103^+^ T cells (TCRβ^+^ CD103^+^) from CD45.1^+^ mice by i.v. injection. On day 7, the spleens, lungs, livers, and kidneys were harvested for analysis of the CD45.1^+^ cell number.

### Whole‐Exome Sequencing

Genomic DNA samples were isolated from the small size of lung tumors and normal lung tissues for mutation detection.^[^ ^80^, ^81^ ^]^ The degradation and contamination of DNA were monitored on 1% agarose gels. Sequencing libraries were generated using the Agilent SureSelect Mouse All Exon V1 kit (Agilent Technologies, CA, USA) following the manufacturer's recommendations and index codes were added to each sample. The clustering of the index‐coded samples was performed on a cBot Cluster Generation System using Hiseq PE Cluster Kit (Illumina). After cluster generation, the DNA libraries were sequenced on the Illumina Hiseq platform at the Novogene Co, Ltd, Beijing, China. FastQC^[^ ^82^ ^]^ version 0.11.8 was used to check the quality of raw sequencing data. Trimmomatic^[^ ^83^ ^]^ version 0.39 was used to trim low‐quality sequences and adapter sequences with the following steps: 1) removing reads with more than 10 nucleotides aligned to the adapter, allowing ≤ 10% mismatches; 2) removing reads with more than 10% uncertain nucleotides; 3) removing reads with more than 50% low quality (Phred quality <5) nucleotides. The variant calling process followed the GATK Best Practices Workflows short variant discovery (SNPs + Indels) pipeline.^[^ ^84^ ^]^ Then the trimmed sequencing reads were mapped to Mus musculus (house mouse) genome assembly GRCm38 (mm10) using BWA^[^ ^85^ ^]^ version 0.7.15. Samtools^[^ ^86^ ^]^ version 1.4 to transform sam to bam and bam sorting. MarkDuplicates and BQSR in GATK version 4.1.7.0 were used for repetitive sequences removal and base quality score recalibration. For comparing the mutation rates between WT and *Med23* ^−/−^ lungs, and between lung adenocarcinomas and adjacent normal tissues in *Med23* ^−/−^ mice, the variants were called in germline mode through the GATK HaplotypeCaller. Then the variants were filtered with criteria: 1) QUAL > 20; 2) DP > 4; 3) MQ > 30; 4) VAF > 0.02. For analyzing the SNV and oncogene mutation in *Med23* ^−/−^ lung adenocarcinomas, somatic mutations were called with Mutect2 in GATK with tumor‐normal mode. The somatic mutation was filtered with “FilterMutectCalls” in GATK, then selected with “PASS” flag and “max‐indel‐size < 10”. ANNOVAR^[^ ^87^ ^]^ version 2019‐10‐24 was used to annotate the region and genetic functions of variants.

### RNA‐Seq, Library Generation, and Bioinformatics Analysis

RNA samples were isolated from lung tumors, and normal lung tissues and sorted AT2 cells, and then were extracted, purified, and checked for integrity using an Agilent Bioanalyzer 2100 (Agilent Technologies, Santa Clara, CA, US). Libraries were generated for sequencing using a NEBNext Ultra RNA Library Prep Kit for Illumina (NEB) and were further sequenced on an Illumina Novaseq platform at the Novogene Co, Ltd, Beijing, China. FastQC^[^ ^82^ ^]^ version 0.11.8 was used to check the quality of raw sequencing data. Trimmomatic^[^ ^83^ ^]^ version 0.39 was used to trim low‐quality sequences and adapter sequences. The trimming criteria for paired reads were the same as in the whole‐exome sequencing. The RNA‐seq reads were aligned to the Mus musculus (house mouse) genome assembly GRCm38 (mm10) using HISAT2^[^ ^88^ ^]^ (v2.0.5). The transcript assembly and quantification were using StringTie^[^ ^89^ ^]^ (v1.3.3b). The gene annotation files (gtf file) were downloaded from the ENSEMBL database. We analyzed these raw count data using DESeq2^[^ ^90^ ^]^ (1.28.1) to assess variance and differential expression between sample groups. Genes with | log_2_ (Fold Change) | ≥ 1 and adjust *p*‐value < 0.05 were defined as differentially expressed genes.

### Single‐Cell RNA‐Seq, Library Generation and Bioinformatics Analysis

Sorted AT2 cell suspensions (2 × 10^5^ cells per mL) were loaded onto a microwell chip using the Singleron Matrix Single Cell Processing System. The mRNA captured by the Barcoding Beads was reverse transcribed, followed by PCR amplification. The scRNA‐seq libraries were generated according to the protocol of the GEXSCOPE Single Cell RNA Library Kits (Singleron) and further sequenced on Illumina Novaseq 6000.^[^ ^91^ ^]^ STAR v2.6.1a was used to map reads to Mus musculus (house mouse) genome assembly GRCm38 (10 mm).^[^ ^92^ ^]^ UMI counts and gene counts of each cell were acquired with featureCounts v2.0.1.^[^ ^93^ ^]^ Seurat v4.04 was used for the downstream analysis.^[^ ^94^ ^]^ Low‐quality cells with more than 20% of transcripts derived from the mitochondria, less than 200 expressed genes, and more than 20000 UMIs were filtered out. The top 2000 highly variable genes were used for the principal component analysis (PCA). Cells were clustered by executing the FindNeighbors and FindClusters functions, and the RunUMAP function was used for clustering visualization.

### Cibersortx Bulk RNA‐Seq Cell Type Deconvolution

The scRNA‐seq dataset from GSE151974^[^ ^23^ ^]^ was used as a cell type reference in Cibersortx,^[^ ^95^ ^]^ and the cell type annotations of adult mouse lung tissues were used.^[^ ^23^ ^]^ Both the scRNA‐seq dataset and the bulk RNA‐seq datasets were inputted in raw count matrices. The signature matrix of the scRNA‐seq dataset was built with the Cibersortx protocol for “scRNA‐seq.” Deconvolution was performed with the “Impute Cell Fractions” module, and the permutations for significance analysis were set as 500. All other Cibersortx parameters were set as default.

### Gene Set Enrichment Analysis

The GSEA^[^ ^96^ ^]^ tool from the Broad Institute website was used to determine enriched pathways. Mouse hallmark gene sets were directly downloaded from the GSEA‐MSigDB database. Gene sets with FDR < 0.25, *p*‐values < 0.05 and | NES | > 1 were defined as significant enrichment pathways.

### Correlation Analysis

The scRNA‐seq of human lung samples from GSE161382^[^ ^55^ ^]^ was used to evaluate the correlation between CD103^+^ T cell frequency in αβ T cells and the accumulation of AT2 cells bearing oxidative stress. The scRNA‐seq was processed with Seurat v4.04 and the αβ T cell sub‐cluster with significant CD103 expression was defined as CD103^+^ T cells.^[^ ^94^ ^]^ The oxidative stress of AT2 cells was evaluated by GO BP term “NEGATIVE_REGULATION_OF_OXIDATIVE_PHOSPHORYLATION” using AUCell v1.12.0. The correlation analysis was calculated by the Spearman rank correlation test.

### Statistical Analysis

All experiments described above were performed at least three times unless otherwise indicated. Statistical analyses were performed using GraphPad Prism6. Data are presented as mean ± s.d. or s.e.m. Paired or unpaired two‐tailed Student's *t*‐test, Wilcoxon Rank Sum test, one‐way ANOVA with Tukey posthoc test, two‐way ANOVA with Bonferroni posthoc test, Fisher's exact test, and Spearman rank correlation test were used to calculate *p* values. For all experiments: ^*^ *p* < 0.05; ^**^ *p* < 0.001; ^***^ *p* < 0.0001, ^****^ *p* < 0.0001.

## Conflict of Interest

The authors declare no conflict of interest.

## Author Contributions

Y.X. and H.L. contributed equally to this work. Y.X. performed most of the work, designed analyzed all experiments, and wrote the manuscript. H.L. performed most of the work and analyzed data. J.W. analyzed WES data, scRNA‐seq data, and RNA‐seq data. H.L. helped with mouse construction and breeding. L.C. and H.J. analyzed data. Z.D. directed the study, and reviewed and edited the manuscript. X.L. conceptualized the research, directed the study, and reviewed and edited the manuscript.

## Supporting information



Supporting Information

## Data Availability

The data that support the findings of this study are available from the corresponding author upon reasonable request.
