# Supplementary material for: CD103+ T Cells Eliminate Damaged Alveolar Epithelial Type II Cells Under Oxidative Stress to Prevent Lung Tumorigenesis
Source: Adv Sci (Weinh). 2025 May 8;12(28):2503557. doi: 10.1002/advs.202503557 (PMC12302525; doi:10.1002/advs.202503557)
Supplement: Supplementary file 1 — Supporting Information [file ADVS-12-2503557-s001.pdf]

ADVANCED SCIENCE
Open Access

Supporting Information

for *Adv. Sci.*, DOI 10.1002/advs.202503557

CD103^{+} T Cells Eliminate Damaged Alveolar Epithelial Type II Cells Under Oxidative Stress to Prevent Lung Tumorigenesis

Yu Xu, Haorui Luo, Jiahao Wang, Haifeng Liu, Luonan Chen, Hongbin Ji, Zimu Deng and Xiaolong Liu**

Supporting Information

CD103$^+$ T cells eliminate damaged alveolar epithelial type II cells under oxidative stress to prevent lung tumorigenesis

Yu Xu, Haorui Luo, Jiahao Wang, Haifeng Liu, Luonan Chen, Hongbin Ji, Zimu Deng, and Xiaolong Liu**

*Correspondence to: dengzimu@simm.ac.cn; liux@sibcb.ac.cn

This PDF file includes:

Figures S1 to S20
Tables S1 to S5

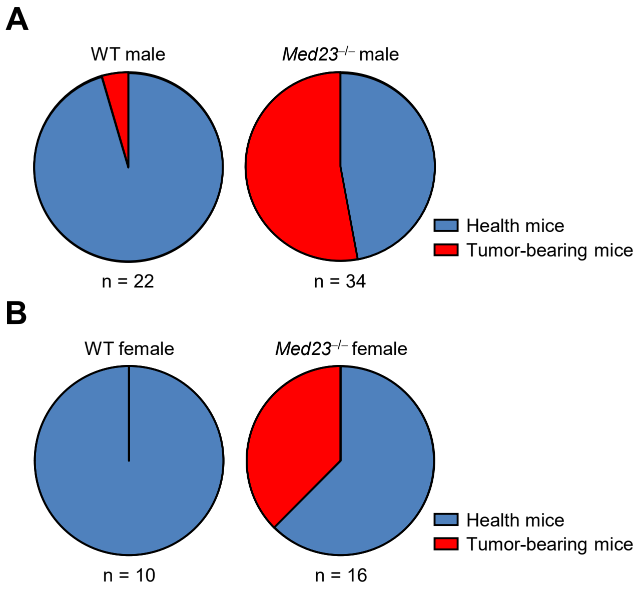

Figure S1. Increased the occurrence of spontaneous tumors in *Med23*$^{-/-}$ aged mice. (**A** and **B**) The occurrence of spontaneous tumors in WT and *Med23*$^{-/-}$ male (**A**) and female (**B**) aged mice (WT male: *n* = 22 mice; *Med23*$^{-/-}$ male: *n* = 34 mice; WT female: *n* = 10 mice; *Med23*$^{-/-}$ female: *n* = 16 mice). All data are combined from (A, B) at least three independent experiments.

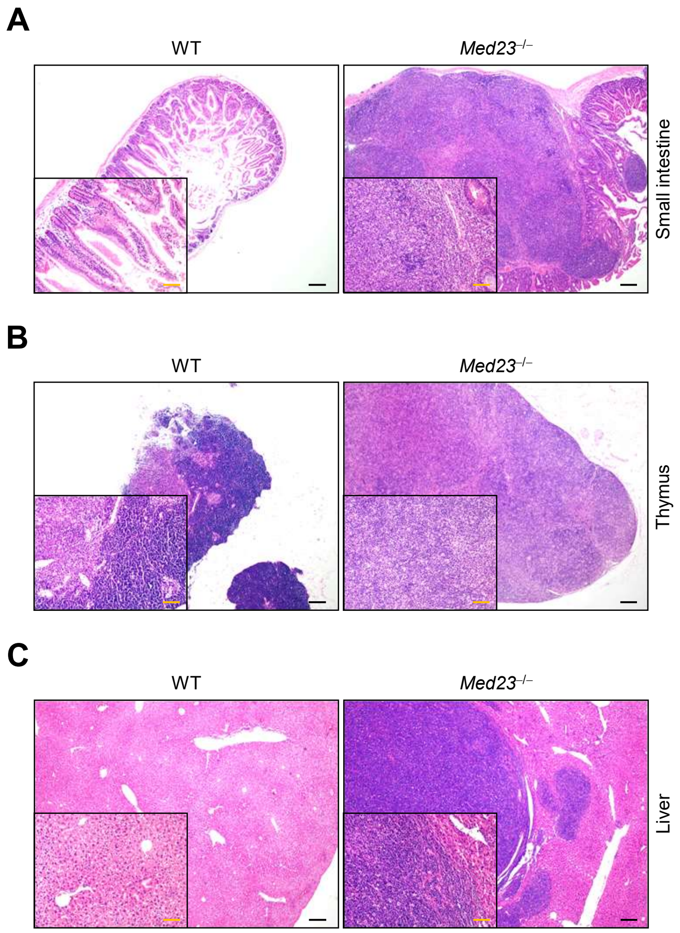

Figure S2. Mice with *Med23* deletion in T cells develop tumors in the small intestine, thymus and liver. (**A**-**C**) Representative H&E staining of the small intestine (**A**), thymus (**B**) and liver (**C**) in WT and *Med23*$^{-/-}$ aged mice. Scale bar: black, 200 μm; orange, 100 μm.

All data are representative of (A-C) at least three independent experiments.

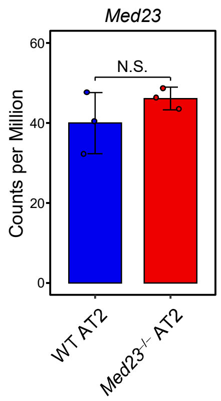

Figure S3. MED23 expression in AT2 cells from WT and *Med23*$^{-/-}$ mice. RNA-seq analysis of *Med23* mRNA levels in AT2 cells from WT and *Med23*$^{-/-}$ mice (n = 3 mice).

The data are presented as the mean ± s.d. For the panel: Student's *t*-test; N.S.: no significance. The data are combined from two independent experiments.

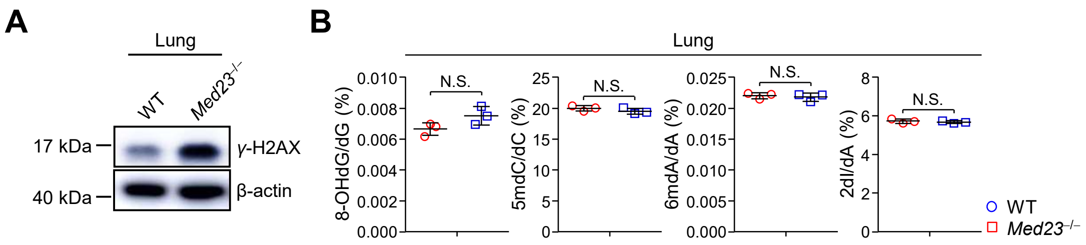

Figure S4. ***Med23*** **deficiency in T cells slightly increases oxidative DNA damage in lungs.** (**A**) Immunoblot analysis of γ-H2AX in lungs from WT and *Med23*$^{-/-}$ aged mice. (**B**) The genomic DNA of lungs was isolated from WT and *Med23*$^{-/-}$ aged mice and was further digested into nucleotides. By HPLC-MS measurement, we analyzed the percentage of 8-OHdG in dG, 5-methyl-2′-deoxycytidine (5mdC) in dC, *N*6-methyl-2′-deoxyadenosine (6mdA) in dA and 2′-deoxyinosine (2dI) in dA (n = 3 mice).

The data (B) are presented as the mean ± s.d. For all panels: Student's *t*-test; N.S.: no significance. All data are representative of (A, B) at least two independent experiments.

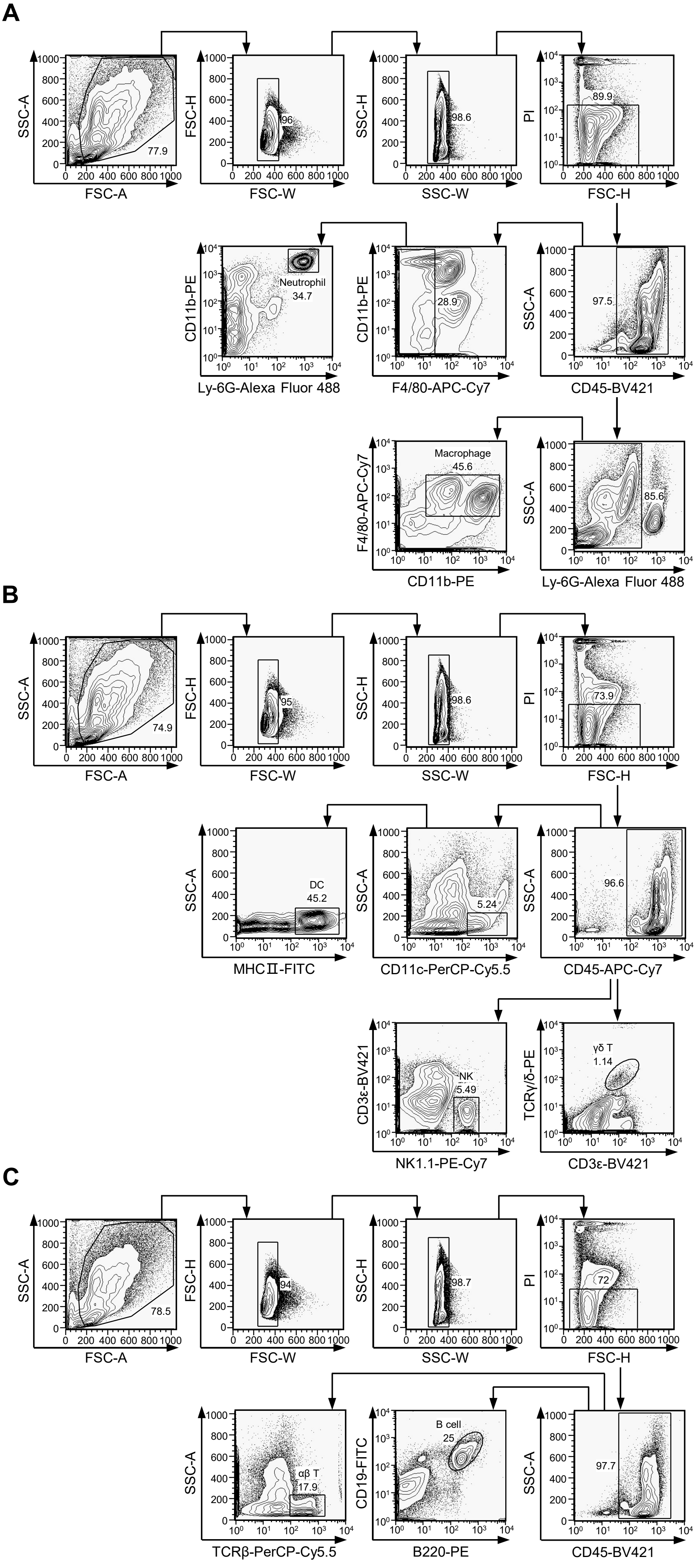
A
SSC-A
FSC-A
77.9
FSC-H
FSC-W
96
SSC-H
SSC-W
98.6
PI
FSC-H
89.9
CD11b-PE
Ly-6G-Alexa Fluor 488
Neutrophil
34.7
F4/80-APC-Cy7
28.9
SSC-A
CD45-BV421
97.5
F4/80-APC-Cy7
CD11b-PE
Macrophage
45.6
Ly-6G-Alexa Fluor 488
85.6
B
74.9
95
98.6
73.9
MHCⅡ-FITC
DC
45.2
CD11c-PerCP-Cy5.5
5.24
CD45-APC-Cy7
96.6
CD3ε-BV421
NK1.1-PE-Cy7
NK
5.49
TCRγ/δ-PE
γδ T
1.14
C
78.5
94
98.7
72
TCRβ-PerCP-Cy5.5
αβ T
17.9
CD19-FITC
B220-PE
B cell
25
97.7

Figure S5. Gating strategies for flow cytometry analysis of neutrophils, macrophages, DCs, NK cells, γδ T cells, αβ T cells and B cells in WT and *Med23*$^{-/-}$ lungs. (**A**) Gating strategy to analyze neutrophils and macrophages in lungs. (**B**) Gating strategy to analyze DCs, NK cells and γδ T cells in lungs. (**C**) Gating strategy to analyze αβ T cells and B cells in lungs.

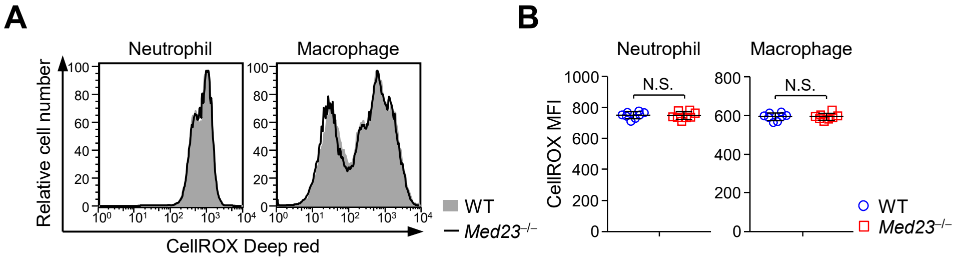

Figure S6. *Med23* deletion in T cells does not influence ROS production of neutrophils and macrophages in lungs. (**A** and **B**) Representative flow cytometry histograms (**A**) and Mean fluorescence intensity (MFI) (**B**) of CellROX in neutrophils and macrophages from WT and *Med23*$^{-/-}$ lungs (n = 8 mice).

The data (B) are presented as the mean ± s.d. For all panels: Student's *t*-test; N.S.: no significance. All data are representative of (A) or combined from (B) at least three independent experiments.

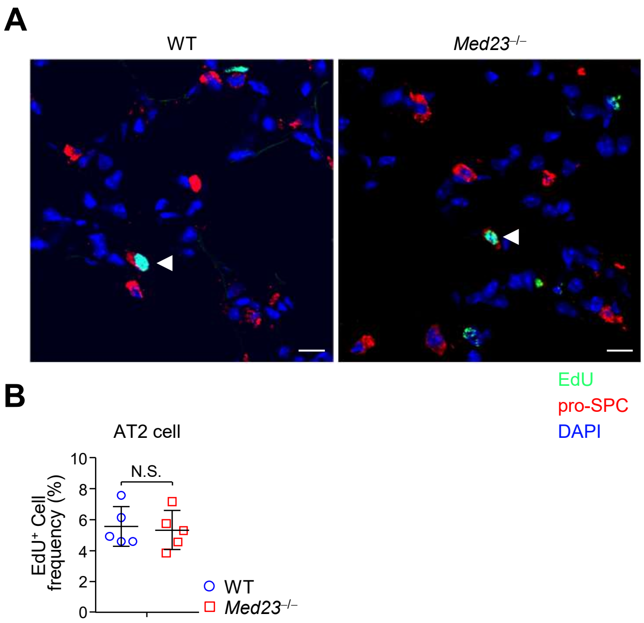

Figure S7. Loss of MED23 in T cells does not impact AT2 cell proliferation. (**A**) Representative immunofluorescence staining for EdU and pro-SPC in lungs of WT and *Med23*$^{-/-}$ mice. Arrowhead indicates EdU^{+} AT2 cells. Scale bar: 10 μm. (**B**) Quantification of the percentage of AT2 cells with incorporated EdU in WT and *Med23*$^{-/-}$ AT2 cells (n = 5 mice).

The data (B) are presented as the mean ± s.d. For all panels: Student's t-test; N.S.: no significance. All data are representative of (A) or combined from (B) at least three independent experiments.

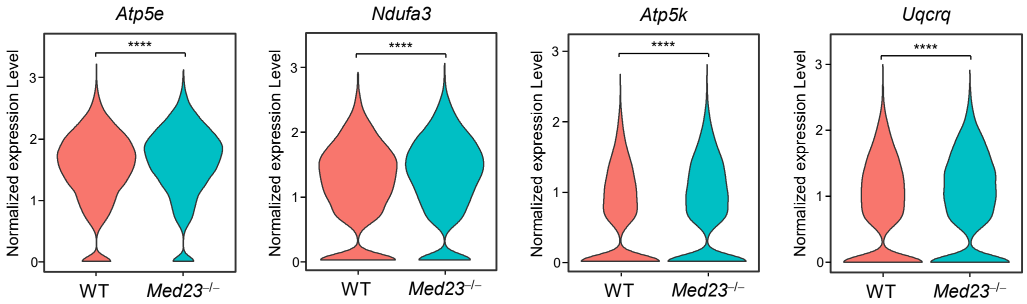

Figure S8. Featuring OXPHOS gene expression in AT2 cells from WT and *Med23*$^{-/-}$ mice. Single-cell RNA sequencing analysis of *Atp5e*, *Ndufa3*, *Atp5k* and *Uqcrq* mRNA levels in AT2 cells from WT and *Med23*$^{-/-}$ mice (WT: $n = 7756$ cells; *Med23*$^{-/-}$: $n = 7807$ cells).

For all panels: ****$P < 0.0001$ by Wilcoxon Rank Sum test. All data are combined from one independent experiments.

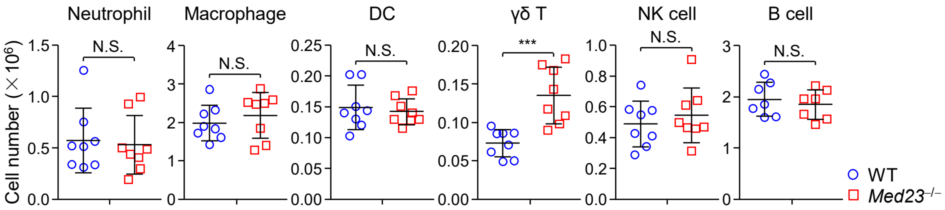

Figure S9. Loss of MED23 in T cells does not impair the absolute number of other immune cells in lungs. The absolute number of neutrophils, macrophages, DCs, γδ T cells, NK cells and B cells in lungs from WT and *Med23*$^{-/-}$ mice (Neutrophil, Macrophage, DC, γδ T and NK cell: n = 8 mice; B cell: n = 7 mice).

The data are presented as the mean ± s.d. For all panels: ***$P < 0.001$ by Student's t-test; N.S.: no significance. The data are combined from at least three independent experiments.

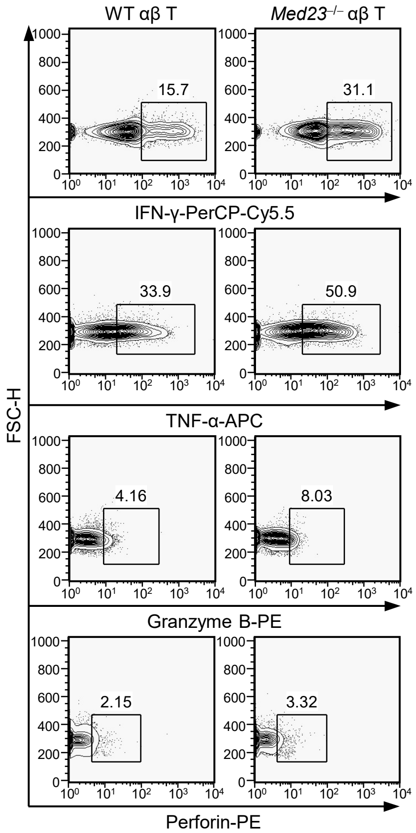

Figure S10. ***Med23****$^{-/-}$* **T cells increase the production of effector cytokines.** Production of IFN-γ, TNF-α, Granzym B and Perforin in WT and *Med23*$^{-/-}$ αβ T cells of lungs after stimulation with PMA (50 ng ml^{-1}) and ionomycin (1 μg ml^{-1}) in the presence of brefeldin A for 3.5 h.

The data are representative of at least three independent experiments.

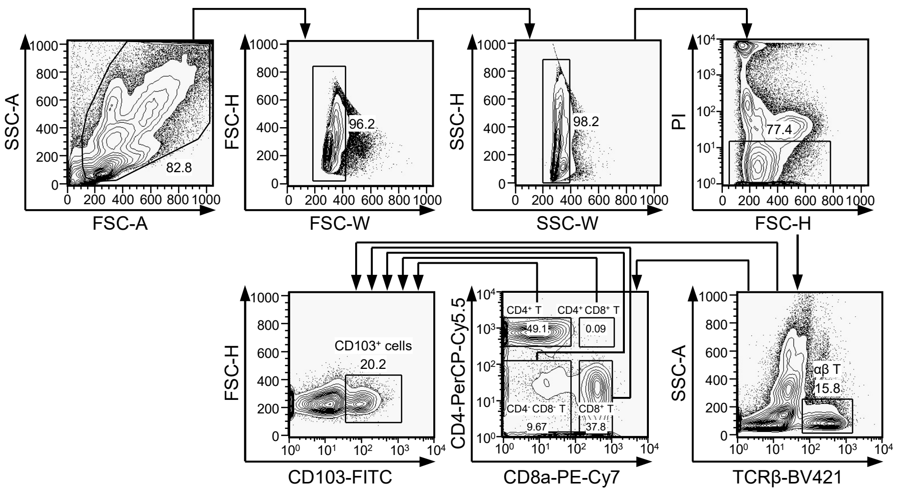

Figure S11. Gating strategies for flow cytometry analysis of CD103 expression in αβ T cells. Gating strategy to analyze CD103^{+} T cells, CD103^{+} CD4^{+} T cells, CD103^{+} CD8^{+} T cells, CD103^{+} CD4^{-} CD8^{-} T cells and CD103^{+} CD4^{+} CD8^{+} T cells in lungs.

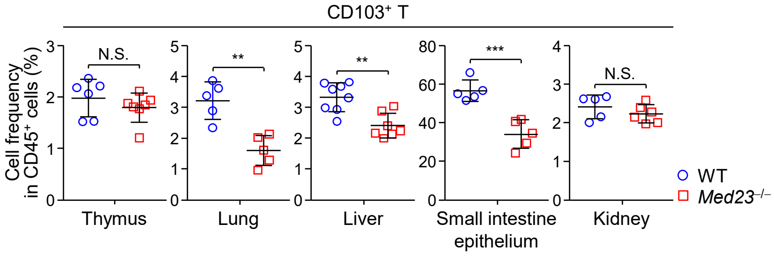

Figure S12. CD103+ T cell enrichment of the lung, liver and small intestine epithelium is impaired in *Med23-/-* mice. The percentage of CD103+ T cells in CD45+ cells from the WT and *Med23-/-* thymus, lung, liver, small intestine epithelium and kidney (Thymus: WT, n = 6 mice, *Med23-/-*, n = 7 mice; Lung and Small intestine epithelium: n = 5 mice; Liver: WT, n = 8 mice, *Med23-/-*, n = 7 mice; Kidney: WT, n = 5 mice, *Med23-/-*, n = 6 mice).

The data are presented as the mean ± s.d. For all panels: **$P < 0.01$; ***$P < 0.001$ by Student's t-test; N.S.: no significance. The data are combined from at least three independent experiments.

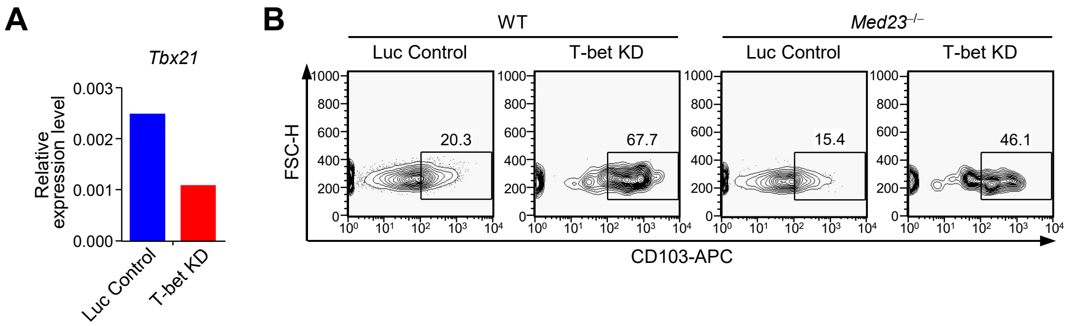

Figure S13. Knocked down T-bet increases the lung *Med23*$^{-/-}$ CD103$^+$ CD8$^+$ T cell generation. (A) Quantitative RT-PCR analysis of *Tbx21* mRNA levels in spleen T cells infected with retrovirus to express luciferase control or T-bet shRNA (*n* = 1 mice). All expression levels were normalized to *Gapdh* expression. (B) Isolated WT or *Med23*$^{-/-}$ spleen-derived naïve T cells were infected with retrovirus to express luciferase control or T-bet shRNA (GFP-labeled). The efficiency of transduction of CD8$^+$ T cells was assessed by GFP expression. *Rag2*$^{-/-}$ mice received 1 $\times$ 10^6 transduced T cells by retro-orbital injection. On days 7, flow cytometric analysis of CD103 expression on lung GFP$^+$ CD8$^+$ T cells with indicated conditions.

All data are representative of (A, B) at least one independent experiments.

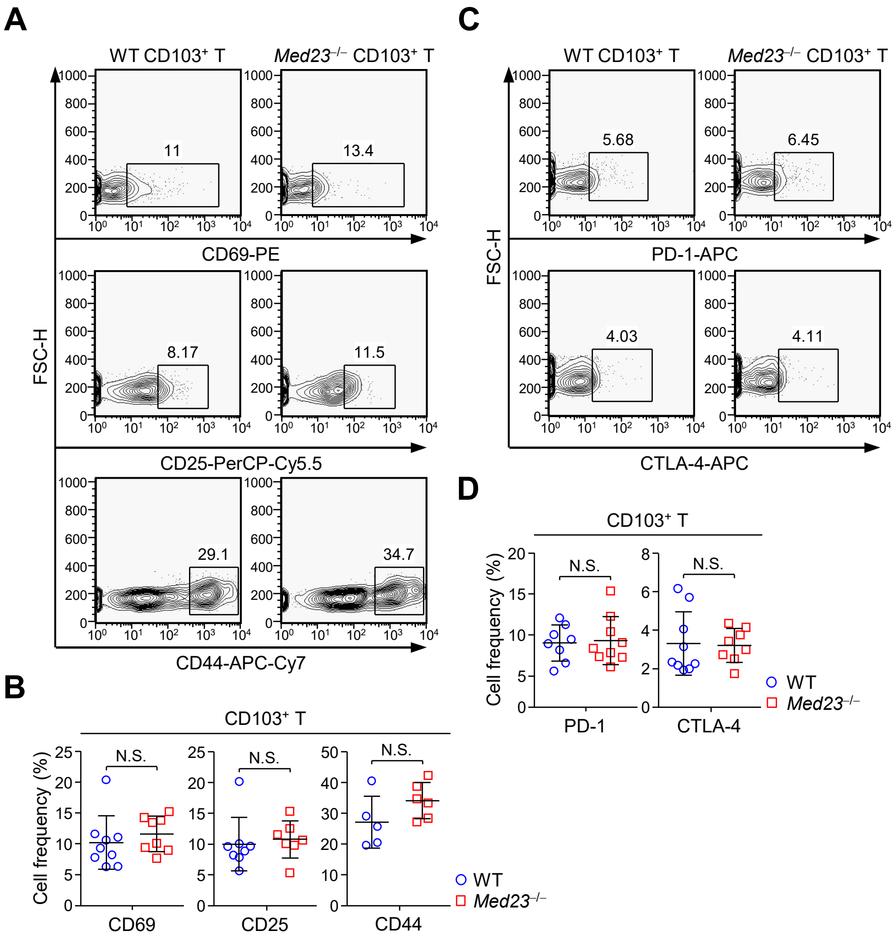

Figure S14. Loss of MED23 does not regulate CD103⁺ T cell activation. (**A**) Flow cytometric analysis of CD69, CD25 and CD44 expression in lung CD103⁺ T cells from WT and *Med23*$^{-/-}$ mice. (**B**) The frequency of CD69⁺ cells, CD25⁺ cells and CD44⁺ cells among CD103⁺ T cells in lungs from WT and *Med23*$^{-/-}$ mice (CD69: WT, $n = 9$ mice, *Med23*$^{-/-}$, $n = 8$ mice; CD25: WT, $n = 8$ mice, *Med23*$^{-/-}$, $n = 7$ mice; CD44: WT, $n = 5$ mice, *Med23*$^{-/-}$, $n = 6$ mice). (**C**) Flow cytometric analysis of PD-1 and CTLA-4 expression in lung CD103⁺ T cells from WT and *Med23*$^{-/-}$ mice. (**D**) The percentage of PD-1⁺ cells and CTLA-4⁺ cells among CD103⁺ T cells in lungs from WT and *Med23*$^{-/-}$ mice (PD-1: WT, $n = 8$ mice, *Med23*$^{-/-}$, $n = 9$ mice; CTLA-4: WT, $n = 9$ mice, *Med23*$^{-/-}$, $n = 8$ mice).

The data (B, D) are presented as the mean ± s.d. For all panels: Student's *t*-test; N.S.: no significance. All data are representative of (A, C) or combined from (B, D) at least three independent experiments.

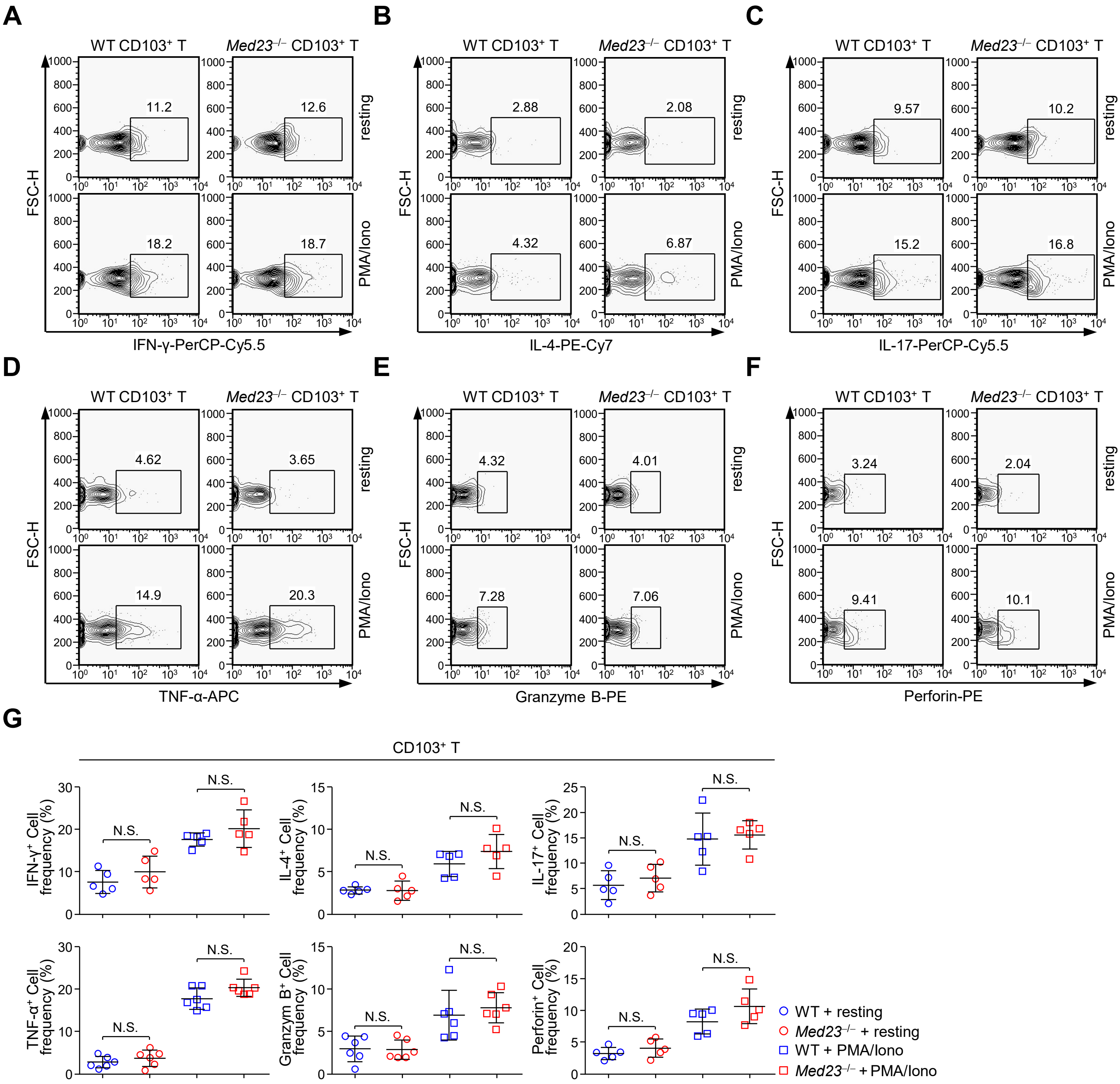

Figure S15. Cytokine secretion of lung CD103⁺ T cells is similar in WT and *Med23*$^{-/-}$ mice. (**A-F**) Production of IFN-γ (**A**), IL-4 (**B**), IL-17 (**C**), TNF-α (**D**), Granzym B (**E**) and Perforin (**F**) in WT and *Med23*$^{-/-}$ CD103^{+} T cells of lungs after stimulation with or without PMA (50 ng ml^{-1}) and ionomycin (1 μg ml^{-1}) in the presence of brefeldin A for 3.5 h. (**G**) The percentage of IFN-γ^{+} cells, IL-4^{+} cells, IL-17^{+} cells, TNF-α^{+} cells, Granzym B^{+} cells and Perforin^{+} cells among CD103^{+} T cells in lungs from WT and *Med23*$^{-/-}$ mice after with or without PMA and ionomycin treatment in the presence of brefeldin A for 3.5 h (IFN-γ, IL-4, IL-17 and Perforin: $n = 5$ mice; TNF-α and Granzym B: $n = 6$ mice).

The data (G) are presented as the mean ± s.d. For all panels: one-way ANOVA with Tukey post-hoc test; N.S.: no significance. All data are representative of (A-F) or combined from (G) at least three independent experiments.

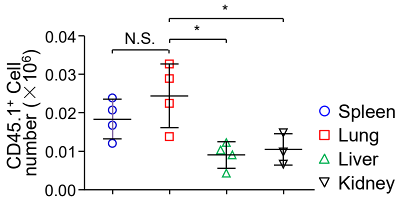

Figure S16. The distribution of transferred CD103^{+} T cells in *Rag2$^{-/-}$* recipient mice. *Rag2$^{-/-}$* mice received 1 × 10^{5} lung CD103^{+} T cells (CD45.1^{+}) by i.v. injection. On days 7, the absolute number of CD45.1^{+} cells in the spleen, lung, liver and kidney of *Rag2$^{-/-}$* recipient mice were analyzed (Spleen, Lung and Liver: $n = 4$ mice; Kidney: $n = 3$ mice).

The data are presented as the mean ± s.d. For the panel: $^{*}P < 0.05$ by one-way ANOVA with Tukey post-hoc test; N.S.: no significance. The data are combined from at least three independent experiments.

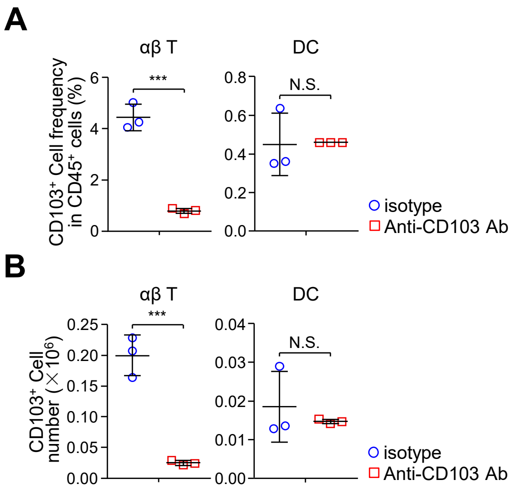

Figure S17. The clearance effect of CD103 neutralizing antibodies. (**A** and **B**) C57BL/6 mice received isotype or CD103 antibody by i.p. injection on days 0. On days 7, the frequency (**A**) and absolutely number (**B**) of CD103$^+$ cells in αβ T cells and DCs were assessed (n = 3 mice).

The data (A, B) are presented as the mean ± s.d. For all panels: ***$P < 0.001$ by Student's t-test; N.S.: no significance. All data are combined from (A, B) at least three independent experiments.

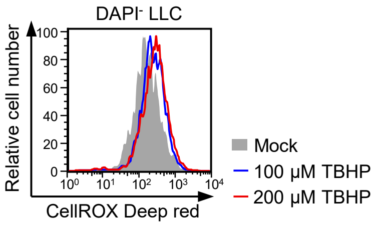

Figure S18. TBHP increase ROS production in LLC cells. Representative flow cytometry histograms of CellROX in LLC cells after treated with mock or indicated dose of TBHP.
The data are combined from at least two independent experiments.

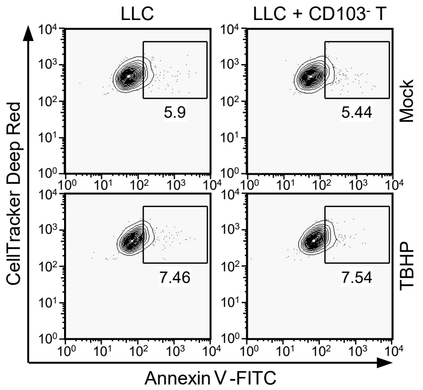

Figure S19. Lung CD103$^-$ T cells do not eliminate the oxidative stressed cells. After treated with mock or TBHP (200 μM), LLC cells either cultured alone or co-cultured with isolated lung CD103$^-$ T cells for 6 hours. Representative flow cytometry analysis of Annexin V expression in DAPI$^-$ LLC cells were displayed.

The data are representative of at least three independent experiments.

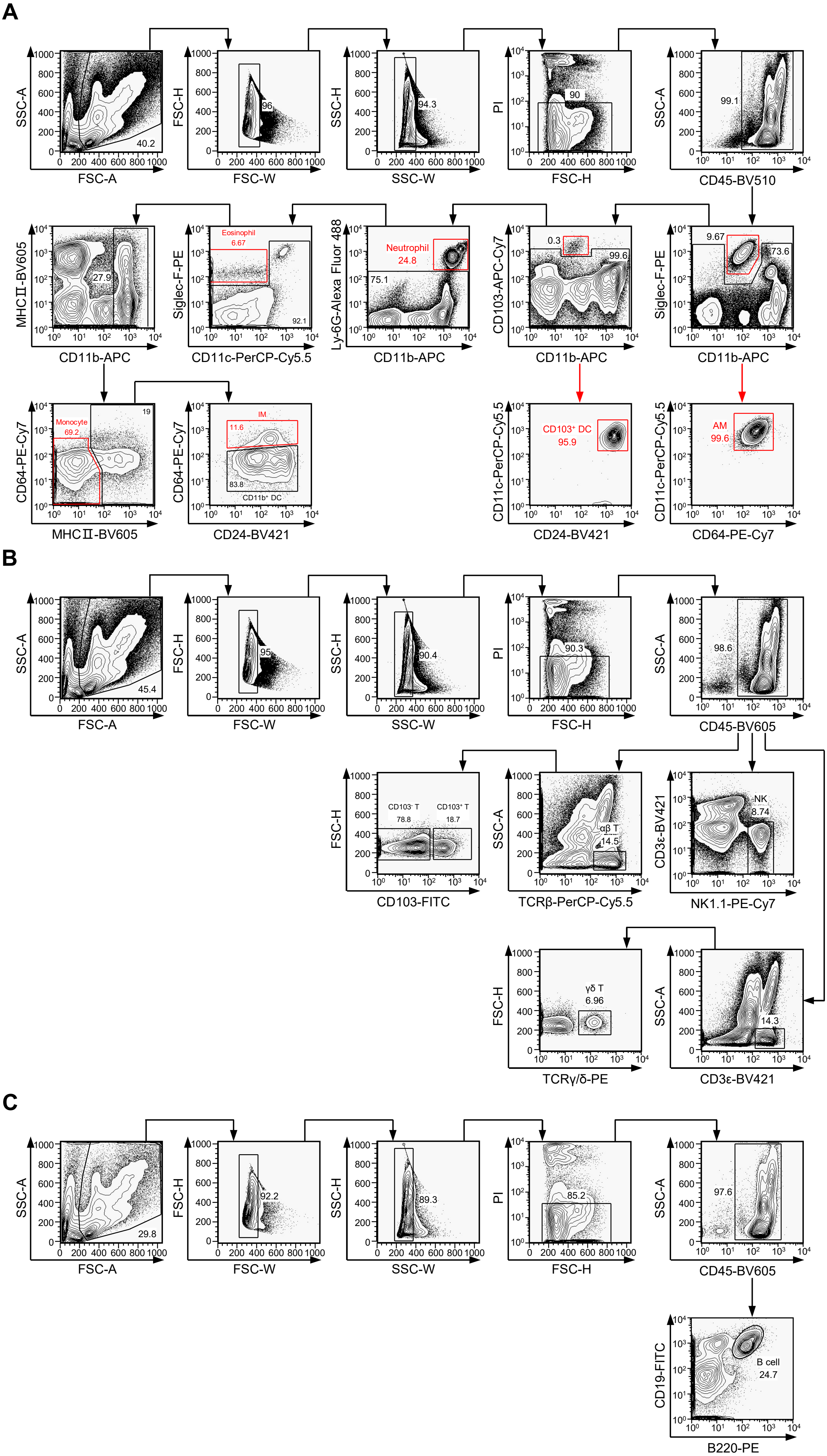
A
SSC-A
FSC-A
40.2
FSC-H
FSC-W
96
SSC-H
SSC-W
94.3
PI
FSC-H
90
SSC-A
CD45-BV510
99.1
MHCⅡ-BV605
CD11b-APC
27.9
Siglec-F-PE
CD11c-PerCP-Cy5.5
Eosinophil
6.67
92.1
Ly-6G-Alexa Fluor 488
CD11b-APC
Neutrophil
24.8
75.1
CD103-APC-Cy7
CD11b-APC
0.3
99.6
Siglec-F-PE
CD11b-APC
9.67
73.6
CD64-PE-Cy7
MHCⅡ-BV605
Monocyte
69.2
19
CD64-PE-Cy7
CD24-BV421
IM
11.6
83.8
CD11b⁺ DC
CD11c-PerCP-Cy5.5
CD24-BV421
CD103⁺ DC
95.9
CD11c-PerCP-Cy5.5
CD64-PE-Cy7
AM
99.6
B
SSC-A
FSC-A
45.4
FSC-H
FSC-W
95
SSC-H
SSC-W
90.4
PI
FSC-H
90.3
SSC-A
CD45-BV605
98.6
FSC-H
CD103-FITC
CD103⁻ T
78.8
CD103⁺ T
18.7
SSC-A
TCRβ-PerCP-Cy5.5
αβ T
14.5
CD3ε-BV421
NK1.1-PE-Cy7
NK
8.74
FSC-H
TCRγ/δ-PE
γδ T
6.96
SSC-A
CD3ε-BV421
14.3
C
SSC-A
FSC-A
29.8
FSC-H
FSC-W
92.2
SSC-H
SSC-W
89.3
PI
FSC-H
85.2
SSC-A
CD45-BV605
97.6
CD19-FITC
B220-PE
B cell
24.7

Figure S20. Gating strategies for flow cytometry analysis of immune cells in lungs from young and aged C57BL/6 mice. (**A**) Gating strategy to analyze alveolar macrophages (AM), interstitial macrophages (IM), monocytes, neutrophils, eosinophils, CD11b$^+$ DCs and CD103$^+$ DCs in lungs. (**B**) Gating strategy to analyze NK cells, CD103$^-$ T cells, CD103$^+$ T cells and γδ T cells in lungs. (**C**) Gating strategy to analyze B cells in lungs.

Table S1. The full name of cell populations in lungs

Abbreviation	Cell type
Alv MΦ	Alveolar macrophages
Art	Arterial endothelial cells
AT1	Alveolar type 1 cells
AT2	Alveolar type 2 cells
pro-B cell	B-cell progenitors
Cap	General capillary endothelial cells
Cap-a	Capillary endothelial cells - aerocytes
Ciliated	Ciliated cells
Club	Club cells
DC1	Dendritic cells 1
DC2	Dendritic cells 2
ILC2	Innate lymphoid cells 2
Int MΦ	Interstitial macrophages
Lymph	Lymphatic endothelial cells
Mast Ba2	Mast basophils 2
Mesothelial	Mesothelial cells
Mono	Monocytes
Neut 1	Neutrophils 1
Neut 2	Neutrophils 2
SMC	Smooth muscle cells
Treg	Regulatory T cells
Vein	Venous endothelial cells

Table S2. Mouse information

Genotype	Strain
Med23$^{fl/fl}$	C57BL/6
Cd4-Cre	C57BL/6
Rag2$^{-/-}$	C57BL/6
Sftpc-DreER	C57BL/6 × ICR
K-ras$^{Rox–Stop–Rox–G12D/+}$	129
Wild type	C57BL/6

Table S3. Primer sequences for genotyping and qPCR

Primer name	Sequence	Application
Cd4-Cre forward	5′-CCTGATCCTGGCAATTTCGG -3′	genotyping
Cd4-Cre reverse	5′-CCCAACCAACAAGAGCTC-3′	genotyping
Med23 forward	5′-GCGGCCGCTATATGCACTGTTAGTGATT-3′	genotyping
Med23 reverse	5′-GTCGACCTTAGAAGAAAGCTCAAACAT-3′	genotyping
Sftpc-DreER forward	5′-AAGCCAAGGACTTGGCTGGC-3′	genotyping
Sftpc-DreER-wt reverse	5′-CTAAGCTAATTGAGTATATGG-3′	genotyping
Sftpc-DreER-mut reverse	5′-ATCTAGCCCAGGAGTGGAAC-3′	genotyping
K-ras-RSR-G12D forward	5′-TGCAGTTTTGACACCAGCTTCG-3′	genotyping
K-ras-RSR-G12D reverse	5′-CTACCCGTGATATTGCTGAAGA-3′	genotyping
Abl1 forward	5′-GCCTCATCACCACACTCCACTAC-3′	qPCR
Abl1 reverse	5′-TTCATCACCGCCGCTTCCTTC-3′	qPCR
Sox9 forward	5′-AACGCACATCAAGACGGAGCAG-3′	qPCR
Sox9 reverse	5′-GGCTGTAGTGAGGAAGGTTGAAGG-3′	qPCR
Egfr forward	5′-TCCTGATTGGTGCTGTGCGATTC-3′	qPCR
Egfr reverse	5′-TCTGGCAGTTCTCCTCTCCTCCT-3′	qPCR
Kit forward	5′-CTCGGACAGCACCAAGCACATT-3′	qPCR
Kit reverse	5′-GTAACCATCACAGAAGCCAGAAGGA-3′	qPCR
Cdkn2a forward	5′-CGCAGGTTCTTGGTCACTGT-3′	qPCR
Cdkn2a reverse	5′-TGTTCACGAAAGCCAGAGCG-3′	qPCR
Trp53 forward	5′-CGGCTCTGAGTATACCACCATCCA-3′	qPCR
Trp53 reverse	5′-TTCTTCTTCTGTACGGCGGTCTCT-3′	qPCR
Npm1 forward	5′-ATGGAAGACTCGATGGATATGGA-3′	qPCR
Npm1 reverse	5′-ACCGTTCTTAATGACAACTGGTG-3′	qPCR
Pdgfra forward	5′-GCCTCGTGCTTGGTCGGATT-3′	qPCR
Pdgfra reverse	5′-GCATCTTCACAGCCACCTTCATTAC-3′	qPCR
Jun forward	5′-GTCCCCTATCGACATGGAGTCT-3′	qPCR
Jun reverse	5′-GGAGTTTTGCGCTTTCAAGGT-3′	qPCR
Fos forward	5′-GGGACAGCCTTTCCTACTAC-3′	qPCR
Fos reverse	5′-GGGATAAAGTTGGCACTAGAG-3′	qPCR
Braf forward	5′-AATTTGGTGGAGAGCATAACCC-3′	qPCR
Braf reverse	5′-ACGGTGTCCATTGATGCAGAG-3′	qPCR
Xiap forward	5′-CGAGCTGGGTTTCTTTATACCG-3′	qPCR
Xiap reverse	5′-GCAATTTGGGGATATTCTCCTGT-3′	qPCR
Cebpa forward	5′-GGTGGACAAGAACAGCAACGAGTA-3′	qPCR
Cebpa reverse	5′-GCGGTCATTGTCACTGGTCAACT-3′	qPCR
Actb forward	5′-TCCGTAAAGACCTCTATGCCAACAC-3′	qPCR
Actb reverse	5′-GTACTCCTGCTTGCTGATCCACAT-3′	qPCR
Tbx21 forward	5′-CAACAACCCCTTTGCCAAAG-3′	qPCR
Tbx21 reverse	5′-TCCCCCAAGCAGTTGACAGT-3′	qPCR
Gapdh forward	5′-CGACTTCAACAGCAACTCCCACTCTTCC-3′	qPCR
Gapdh reverse	5′-TGGGTGGTCCAGGGTTTCTTACTCCTT-3′	qPCR

Table S4. Fluorescently conjugated protein or antibodies used for flow cytometry

Target	Fluorochrome	Clone	Manufacturer	Catalog	Dilution ratio (vol/vol)
Cell surface stain					
TCRβ	APC	H57-597	eBioscience	17-5961-83	1:150
TCRβ	BV421	H57-597	BioLegend	109229	1:150
TCRβ	PerCP-Cy5.5	H57-597	BD	560657	1:150
PD-1	APC	J105	eBioscience	17-2799-42	1:150
CTLA-4	APC	UC10-4B9	eBioscience	17-1522-82	1:150
CD25	PerCP-Cy5.5	PC61	BD	551071	1:150
CD4	PerCP-Cy5.5	RM4-5	BD	550954	1:150
CD4	APC-Cy7	GK1.5	BD	552051	1:150
CD8α	PE-Cy7	53-6.7	BD	552877	1:150
CD8α	APC	53-6.7	eBioscience	17-0081-83	1:150
CD69	PE	H1.2F3	BioLegend	104507	1:150
CD103	FITC	2E7	BioLegend	121419	1:150
CD103	APC	2E7	eBioscience	17-1031-82	1:60
CD103	APC-Cy7	2E7	BioLegend	121431	1:60
CD44	APC-Cy7	IM7	BioLegend	103027	1:150
Ly-6G	AF488	1A8	BioLegend	127626	1:300
CD11b	PE	M1/70	BioLegend	101207	1:150
CD11b	APC	M1/70	BioLegend	101212	1:150
CD11c	PerCP-Cy5.5	N418	eBioscience	45-0114-82	1:150
F4/80	APC-Cy7	BM8	BioLegend	123118	1:150
CD45	BV421	30-F11	BioLegend	103133	1:150
CD45	APC-Cy7	30-F11	BioLegend	103116	1:150
CD45	FITC	30-F11	BioLegend	103107	1:150
CD45	BV510	30-F11	BioLegend	103137	1:150
CD45	BV605	30-F11	BioLegend	103139	1:150
TCRγ/δ	PE	GL3	BioLegend	118108	1:150
CD3ε	BV421	145-2C11	BD	562600	1:150
CD45.1	PE-Cy7	A20	BioLegend	110729	1:150
MHCII	FITC	2G9	BD	553623	1:150
MHCII	BV605	M5/114.15.2	BD	563413	1:150
NK1.1	PE-Cy7	PK136	BD	552878	1:150
Siglec-F	PE	E50-2440	BD	552126	1:150
CD64	PE-Cy7	X54-5/7.1	BioLegend	139314	1:150
CD24	BV421	M1/69	BioLegend	101826	1:150
CD19	FITC	1D3	BD	553785	1:150
B220	PE	RA3-6B2	BD	553090	1:150
Annexin V	FITC		BioLegend	640906	1:20
Intracellular stain					
IFN-γ	PerCP-Cy5.5	XMG1.2	BioLegend	505822	1:150
IL-17	PerCP-Cy5.5	eBio17B7	eBioscience	45-7177-82	1:150
TNF-α	APC	MP6-XT22	BD	554420	1:150
Granzyme B	PE	NGZB	eBioscience	12-8898-80	1:150
Perforin	PE	eBioOMAK-D	eBioscience	12-9392-80	1:150
Ki67	FITC	SolA15	eBioscience	11-5698-82	1:60
T-bet	PE-Cy7	4B10	BioLegend	644823	1:30
IL-4	PE-Cy7	11B11	BioLegend	504117	1:150

AF, Alexa Fluor; APC, allophycocyanin; BV, brilliant violet; FITC, fluorescein isothiocyanate; PE, phycoerythrin; PerCP, peridinin–chlorophyll–protein.

Table S5. The MRM transitions and optimal parameters for the analysis of the DNA nucleosides by HPLC-MS/MS.

Full name	Precursor ion (m/z)	Product ion (m/z)	Collision energy (volt)
Deoxyadenosine	252	136	15
Deoxycytidine	228	112	15
Deoxyguanosine	268	152	15
*N*6-methyl-2′-deoxyadenosine	266	150	20
2′-deoxyinosine	253	137	25
5-methyl-2′-deoxycytidine	242	126	25
8-hydroxy-2'-deoxyguanosine	284	168	10
